# Transcutaneous Electrical Acupoint Stimulation for the Prevention of Postoperative Cognitive Dysfunction: A Systematic Review and Meta-Analysis

**DOI:** 10.3389/fmed.2021.756366

**Published:** 2021-12-06

**Authors:** Tiantian Zhang, Liang Ou, Zehua Chen, Jiamin Li, Yan Shang, Guoheng Hu

**Affiliations:** ^1^Department of Neurology, The First Hospital of Hunan University of Chinese Medicine, Changsha, China; ^2^The Graduate School, Hunan University of Chinese Medicine, Changsha, China; ^3^Department of Orthopedics, The Second Affiliated Hospital of Guizhou University of Traditional Chinese Medicine, Guiyang, China; ^4^The Fifth Clinical Medical College, Guangzhou University of Chinese Medicine, Guangzhou, China

**Keywords:** transcutaneous electrical acupoint stimulation, postoperative cognitive dysfunction, prevention, cognitive function, systematic review, meta-analysis

## Abstract

**Background:** No specific treatment is available for postoperative cognitive dysfunction (POCD). Recently, interest in the prevention of POCD during the perioperative period has increased. Although some studies suggest that transcutaneous electrical acupoint stimulation (TEAS) may be beneficial, the relevant evidence remains uncertain.

**Objective:** To evaluate the preventive effects of TEAS on POCD.

**Methods:** Seven databases including PubMed, EMBASE, CENTRAL, China National Knowledge Infrastructure (CNKI), Chinese Scientific Journal Database (VIP), Wanfang Database, and Chinese Biomedical Literature Database (CBM) were electronically searched up to April 2021. Two reviewers independently selected the studies, collected data, and assessed the risks of bias and grading of recommendations, assessment, development, and evaluations certainty of the evidence. A meta-analysis of the incidence of POCD, cognitive function score, pain, adverse reactions, and length of hospital stay after surgery was also performed.

**Results:** Twenty-nine randomized controlled trials with 1,994 participants were included. The results of the meta-analysis showed that the TEAS group has a significantly lower incidence of POCD compared with the control group on postoperative days 1 [OR = 0.33 (95%CI: 0.23, 0.47); *p* < 0.001, *I*^2^ = 0%, moderate certainty], 3 [OR = 0.38 (95%CI: 0.29, 0.50); *p* < 0.001, *I*^2^ = 0%, low certainty], and 7 [OR = 0.51 (95%CI: 0.32, 0.81); *p* = 0.005, *I*^2^ = 0%, low certainty] but not on day 5 (*p* > 0.05, low certainty). Moreover, TEAS improved the Mini-Mental State Examination scores on postoperative days 1, 3, and 7 [MD = 2.44 (95%CI: 1.61, 3.27); *p* < 0.001, *I*^2^ = 93%, low certainty]; [MD = 2.07 (95%CI: 1.53, 2.62); *p* < 0.001, *I*^2^ = 87%, low certainty]; and [MD = 0.49 (95%CI: 0.18, 0.79); *p* = 0.002, *I*^2^ = 21%, low certainty], respectively, but not on day 5 (*p* > 0.05, very low certainty). TEAS promoted a postoperative analgesic effect within 24 h after surgery. Furthermore, patients receiving TEAS showed a lower incidence of postoperative nausea and vomiting and a shorter hospital stay.

**Conclusions:** Limited evidence suggests that the application of TEAS in the perioperative period is associated with a reduced POCD rate and a protected early postoperative cognitive function.

## Introduction

Postoperative cognitive dysfunction (POCD) is a syndrome with prolonged cognitive impairment, which is characterized by limitations in memory, intellectual ability, and executive function after surgery. This condition is distinct from delirium and dementia (1). An estimated 312.9 million surgical procedures were performed worldwide in 2012, displaying an increase of 38% over the previous 8 years (2). Furthermore, with growing populations and increasing lifespan, the number of surgeries performed annually is likely to continue to increase (3). Cognitive dysfunction following a surgical procedure is one of the most common complications in the elderly, with estimated incidence rates of 20–50% at 3 months post-cardiac surgery and 5–55% after other major surgeries (4, 5). A higher 1-year mortality rate increased length of stay and cost, and premature withdrawal from the workforce have been observed in patients who experienced POCD. POCD is also associated with an increased risk of dementia and may lead to chronic neurodegeneration, particularly in the case of repeated surgery (6–8). Age, education level, infection, and preexisting cognitive disorders have been associated with cognitive decline after surgical procedures (4, 7, 9, 10). To date, there remains no available strategy for the treatment of POCD. Therefore, anesthesiologists and surgeons have sought to develop an approach to reduce the morbidity of POCD. As the occurrence of POCD may be multifactorial, the prevention methods are correspondingly multidisciplinary (8, 11, 12).

Acupuncture, as traditional alternative medicine, has been used to treat diseases and relieve pain for thousands of years in Asia. Transcutaneous electrical acupoint stimulation (TEAS) is a combination of transcutaneous electrical nerve stimulation (TENS) and traditional Chinese acupuncture that has been widely accepted and applied worldwide (13). Previous studies have shown the beneficial effects of TEAS for various aspects, such as reducing intraoperative opioid use and postoperative nausea and vomiting (PONV), relieving pain, and improving postoperative cognitive function (14–17). To our knowledge, few studies have systematically examined the efficacy and safety of TEAS for the prevention of POCD based on the Preferred Reporting Items for Systematic reviews and Meta-analyses (PRISMA) guidelines. Therefore, we evaluated the preventive effect of TEAS on POCD to provide evidence for clinical practice by reviewing all currently available randomized controlled trials (RCTs).

## Methods

This systematic review and meta-analysis are reported in accordance with the PRISMA guidelines (18) and registered on the Open Science Framework (https://osf.io/bq4v2, https://doi.org/10.17605/OSF.IO/BQ4V2).

### Databases and Search Strategy

We searched PubMed, EMBASE, Cochrane Central Register of Controlled Trials (CENTRAL), China National Knowledge Infrastructure (CNKI), Chinese Scientific Journal Database (VIP), Wanfang Database, and Chinese Biomedical Literature Database (CBM) from the dates of inception of the databases until April 2021 without any language restrictions. The search strategy used medical subject's headings (MeSH) terms in combination with free-text, such as “Cognitive Function,” “Cognitive Dysfunction,” “Cognition,” “Cognitive Impairments,” “Postoperative Cognitive Complications,” “Transcutaneous Electrical Acupoint Stimulation,” “Acupuncture Points,” and “Electric Stimulation,” etc. The detailed search strategy is described in Supplementary File 1.

### Selection Criteria

This review focused on RCTs that were less prone to confounding bias by indication (19, 20). The eligibility criteria for study selection were as follows: Participants aged 18 years and above who underwent surgery and anesthesia and showed no abnormal cognitive function in preoperative assessment; the intervention group was treated with TEAS, alone or combined with other therapies; the control group (CG) was treated with a sham intervention, with no treatment, or other therapies; and no limit to the type of operation. We excluded studies with participants diagnosed with POCD, trials that used needles, such as electroacupuncture, body needle, auricular needle, etc., and articles published using the same data sets.

### Outcome Measures

The primary outcomes were the incidence of POCD and cognitive function scores assessed by any definition given in the original study. The secondary outcomes included postoperative pain, adverse reactions, and length of hospital stay.

### Data Extraction and Quality Assessment

Two investigators (JML and YS) independently extracted data from eligible studies and inputted the outcome data into a predesigned spreadsheet. Any disagreements in the crosschecking process were resolved through discussion. Otherwise, a third investigator (LO) arbitrated the dispute. The main information extracted from the included articles included study design, populations studied, type of operation, type of anesthesia, intervention, outcomes, and postoperative test time. The Cochrane risk of bias tool was used to assess the methodological quality and the risks of bias of the individual studies (21). The certainty of the evidence for each outcome was evaluated using the grading of recommendations, assessment, development, and evaluations framework (22).

### Data Synthesis and Analysis

Data were synthesized using RevMan version 5.4. For dichotomous outcomes, such as the incidence of POCD, we calculated the odds ratio (OR) and 95% CI. For continuous outcomes, such as cognitive function scores, we pooled the mean difference (MD) and 95% CI. Statistical significance was set at *p* < 0.05.

### Subgroup Analyses and Investigation of Heterogeneity

When sufficient studies reported relevant characteristics, we performed subgroup analyses for postoperative test time, anesthesia method, type of operation, and acupoint combination. We also conducted subgroup analyses to explore the impact of small sample-sized studies by grouping their sample size by quarter (from quarter 1, which includes 25% of the smallest trials, to quarter 4, which includes 25% of the largest trials) (23). The heterogeneity among the studies was assessed using Cochran Q tests (χ^2^ tests for heterogeneity), and significant statistical heterogeneity was defined as a Q test with *p* < 0.10 or *I*^2^ > 50%. We used the random-effects model to calculate the effect size, allowing for differences between the studies (24).

### Publication Bias and Sensitivity Analyses

We tested for publication bias when sufficient studies were available (*n* ≥ 10). Asymmetric funnel and Harbord tests were applied to assess for potential publication bias when OR was used as an effect estimate, and the results showed there was no substantial heterogeneity between the studies. Moreover, when MD was regarded as an effect estimate, we adopted an asymmetric funnel and Egger's test to assess for potential publication bias (25). We also used the trim-and-fill method to identify and correct funnel plot asymmetries caused by publication bias (26). We conducted a sensitivity analysis to explore the source of heterogeneity by removing 1 study in each turn, and to examine the stability of the main outcome by excluding poor-quality trials with high risks of bias.

## Results

### Study Selection and Characteristics

**Figure 1:**
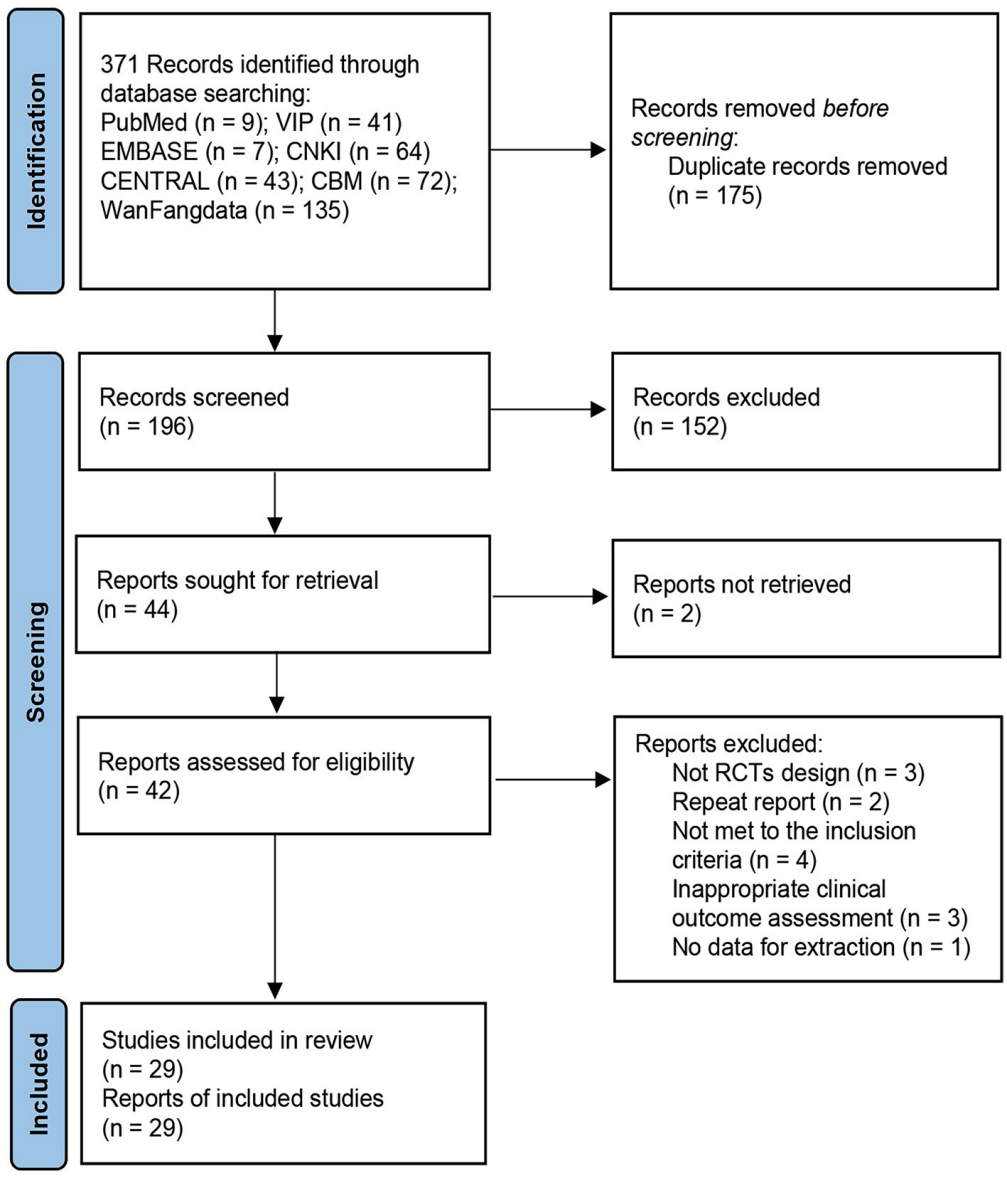
Flowchart of study selection.

The database search identified a total of 371 articles. According to the inclusion and exclusion criteria, 29 RCTs (27–55) with 1,994 participants were eligible for data extraction. A flow diagram of the screening of the trials is shown in Figure 1. In the present review, all trials were conducted in China and published in Chinese and English. The average age of the participants in 11 trails (27, 28, 35, 36, 39, 40, 43, 44, 46, 47, 51) was 18–65 years and was > 65 years in the other studies. Of the 29 reported interventions, 3 trails (28, 32, 50) were treated with a combination of TEAS and controlled hypotension, and 1 (52) received TEAS combined with dexmedetomidine. Of 29 RCTs, 2 trials (27, 39) reported patients who underwent craniocerebral surgery, and 2 other studies (48, 53) involved patients undergoing cardiac surgery. Participants from the remaining 25 trails underwent non-cardiac surgery. The time points for evaluation in the included studies ranged from 0 to 7 days after surgery (Table 1).

**Table 1:** Characteristics of the included randomized clinical trials.

**First author**	**Age (years) EG/CG**	**Sample size**	**Type of operation/anesthesia**	**Intervention**			**Outcomes**	**Postoperative test time**
				**EG**	**CG**	**Acupoints/Stimulation frequency/time**		
Ren (27)	52 ± 9/ 54 ± 10	50	Craniotomy/ Intravenous-inhalation	TEAS	No treatment	LI4, LI11, ST36, SP6; 2~100 Hz; from 30 min before anesthesia to the completion of operation	MMSE	1, 24, 48 h
Zhou et al. (28)	45~65	75	Single spinal surgery/Intravenous-inhalation	TEAS+ Controlled hypotension	Controlled hypotension	LI4, LI11, ST36, SP6; 2~100 Hz; from 30 min before anesthesia to the completion of operation	incidence of POCD	1, 3, 5d
Lin et al. (29)	68.5 ± 2.8/ 67.3 ± 2.7	49	Abdominal surgery/ Intravenous	TEAS	No treatment	GV20, GV29, PC6; 2~100 Hz; from 30 min before anesthesia to the completion of operation	incidence of POCD, MMSE	3d
Wu et al. (30)	62~76	100	Laparoscopic cholecystectomy/ Intravenous-inhalation	TEAS	No treatment	GV20, PC6, ST36; 2~15 Hz; from 30 min before anesthesia to the completion of operation	incidence of POCD and PONV	3, 7d
Ni et al. (31)	65~78	60	Laparoscopic resection of rectal cancer/Intravenous	TEAS	No treatment	GV20, PC6, ST36, SP6; 2~100 Hz; from 30 min before anesthesia to the completion of operation	incidence of POCD, MMSE	1, 3, 5, 7d
Yin et al. (32)	78.3 ± 5.5/ 77.5 ± 5.2	53	Hip-replacement surgery/ Intravenous	TEAS+ Controlled hypotension	Sham stimulation+ Controlled hypotension	GV20, PC6, GB20; 2~100 Hz; from 30 min before anesthesia to the completion of operation	incidence of POCD, MMSE	72 h
Yang et al. (33)	65~80	60	Gynecological laparoscopic surgery/Intravenous	TEAS	Sham stimulation	PC6, ST36; 2~100 Hz; from 30 min before anesthesia to the completion of operation	incidence of POCD, MMSE	1, 3, 5d
Wang(a) et al. (34)	69.9 ± 4.2/ 69.3 ± 4.1	60	Artificial femoral head replacement/CSEA	TEAS	No treatment	GV20, PC6, ST36, SP6; 2~100 Hz; after anesthesia to the end of operation	incidence of POCD, MMSE, VAS	1, 3, 7d
Zhu et al. (35)	34.2 ± 9.7/ 34.6 ± 8.4	60	Gynecological laparoscopic surgery/Intravenous-inhalation	TEAS	No treatment	PC6, ST36; 2~100 Hz; from 30 min before anesthesia to the completion of operation	incidence of POCD, MMSE	1d
Wei et al. (36)	55.2 ± 6.1/ 56.5 ± 4.6	40	Gynecological laparoscopic surgery/Intravenous-inhalation	TEAS	No treatment	GV20, PC6, GB20; 2~100 Hz; 30 min before anesthesia	incidence of POCD, MMSE	1, 3d
Li et al. (37)	65.7 ± 6.1/ 66.5 ± 4.0	60	Radical thoracoscopic lung cancer operation/ Intravenous-inhalation	TEAS	No treatment	PC6, ST36; 2~100 Hz; from 30 min before anesthesia to the completion of operation	incidence of POCD, MMSE	1, 3d
Wang(b) et al. (38)	70.3 ± 4.2/ 69.5 ± 4.4	60	Laparoscopic radical gastrectomy for cancer/ Intravenous	TEAS	No treatment	GV20, PC6, ST36, SP6; 2~100 Hz; after anesthesia to the end of operation	incidence of POCD, MMSE, VAS	1, 3, 7d
Zhao et al. (39)	37.0 ± 11.7/ 37.9 ± 11.9	80	Transsphenoidal surgery/ Intravenous-inhalation	TEAS	No treatment	LI4, TE5, EX-HN4; 2~100 Hz; from 30 min before anesthesia to the completion of operation	incidence of POCD, neuropsychological tests	3d
Wei et al. (40)	57.9 ± 3.9/ 57.6 ± 4.1	40	Gynecological laparoscopic surgery/ Intravenous-inhalation	TEAS	No treatment	GV20, PC6, GB20; 2~100 Hz; 30 min before anesthesia, stimulation for 1 h	incidence of POCD, MMSE	1, 3d
Tan et al. (41)	67.1 ± 6.2/ 66.4 ± 5.5	70	Laparoscopic cholecystectomy/ NR	TEAS	Sham stimulation	GV20, PC6, ST36, SP6; 2~100 Hz; 30 min/d from the first day before operation to the 7 day after operation	incidence of POCD, MMSE	3, 7d
Tang et al. (42)	69.6 ± 5.8/ 70.1 ± 6.3	90	Colorectal cancer surgery/ intravenous	TEAS	No treatment	GV20, GV24; 2~100 Hz; from 30 min before anesthesia to the completion of operation	incidence of POCD, MMSE	1, 3, 5, 7d
Fan et al. (43)	54 ± 7/ 54 ± 8	56	Laparoscopic resection of colorectal cancer/ Intravenous-inhalation	TEAS	No treatment	PC6, LI4, ST36, ST37, ST39; 2~100 Hz; from 30 min before anesthesia to the completion of operation	incidence of POCD and PONV, QoR-15, length of hospital stay	3d
Mi et al. (44)	44 ± 6/ 45 ± 8	100	Laparoscopic cholecystectomy/ Intravenous-inhalation	TEAS	Sham stimulation	LI4, PC6, ST36; 2~100 Hz; from 30 min before anesthesia to the completion of operation	QoR-40, MMSE, incidence of PONV	4, 8, 24, 48 h
Sun et al. (45)	86.3 ± 4.4/ 85.8 ± 4.2	40	Hip fracture surgery/CSEA	TEAS	Sham stimulation	GV20, GB20; 2~100 Hz; (3 times/d, 30 min/time) since 2 days before operation until the operation finished	incidence of POCD, MMSE	24, 72 h
Mao et al. (46)	35~55	80	Breast cancer surgery/ Intravenous	TEAS	No treatment	LI4, PC6; 2~100 Hz; from 10 min before anesthesia to the completion of operation	MMSE, incidence of PONV	24, 48 h
Li and Yang (47)	39.0 ± 5.2/ 39.0 ± 5.3	90	Laparoscopic Myomectomy/ Intravenous-inhalation	TEAS	Dex	PC6, ST36; 2~100 Hz; from 30 min before anesthesia to the completion of operation	incidence of POCD and PONV	3d
Wu and Chen (48)	72.3 ± 5.3/ 71.9 ± 5.1	84	Cardiac surgery/ Intravenous	TEAS	No treatment	GV20, PC6, ST36, SP6; 2~100 Hz; from 30 min before anesthesia to the completion of operation	MMSE, VAS, incidence of PONV	3d
Duan et al. (49)	78 ± 10/ 76 ± 11	80	Hip replacement/ Intravenous-inhalation	TEAS	Sham stimulation	LI4, PC6; 2~200 Hz; from 30 min before anesthesia to the completion of operation	incidence of POCD, MMSE, VAS	24, 72 h
Lu et al. (50)	72.1 ± 2.5/ 71.3 ± 2.3	91	Hip replacement/ Intravenous-inhalation	TEAS+ Controlled hypotension	Controlled hypotension	GV20, PC6, GB20; 2~100 Hz; Before anesthesia induction to the end of operation	incidence of POCD and PONV, MMSE, VAS	72 h
Yu et al. (51)	48.5 ± 16.2/ 45.9 ± 17.5	60	Gynecological laparoscopic surgery/ Intravenous	TEAS	Sham stimulation	GV20, GV29, ST36, PC6; 2~100 Hz; 30 min before anesthesia	QoR-40, MMSE, VAS, incidence of PONV	1, 2d
Yang (52)	71.1 ± 5.4/ 71.0 ± 5.6	60	Hip surgery/CSEA	TEAS+ Dex	Sham stimulation+ Dex	EX-HN3, GB20; 2~100 Hz; from 30 min before anesthesia to the completion of operation	MMSE	24h
Huang et al. (53)	65.3 ± 6.9/ 65.4 ± 5.2	82	Off-pump coronary artery bypass grafting/ Intravenous	TEAS	Sham stimulation	PC6, LI4, GV14; 2~100 Hz; from the beginning of operation to the completion of operation	incidence of POCD, MMSE	7d
Wu and Luo (54)	72 ± 10/ 72 ± 9	84	Non cardiac surgery/ Intravenous	TEAS	Sham stimulation	LI4, PC6; 2~60 Hz; before anesthesia to the completion of operation	incidence of POCD and PONV, MMSE, MoCA	1, 3, 7d
Duan et al. (55)	69.2 ± 7.7/ 70.4 ± 3.9	80	Total hip replacement/ Intravenous	TEAS	Sham stimulation	LI4, PC6; 30 min before anesthesia	incidence of POCD, MMSE, VAS	1, 3d

*EG, experimental group; CG, control group; CSEA, combined spinal epidural anesthesia; TEAS, transcutaneous electrical acupoint stimulation; MMSE, mini-mental state examination; POCD, postoperative cognitive dysfunction; PONV, postoperative nausea and vomiting; VAS, visual analog scale; MoCA, montreal cognitive assessment; QoR-40/15, quality of recovery-40/15; LOTCA, loeweistein occupational therapy cognitive assessment; Dex, Dexmedetomidine; NR, not reported*.

### Quality Assessments

**Figure 2:**
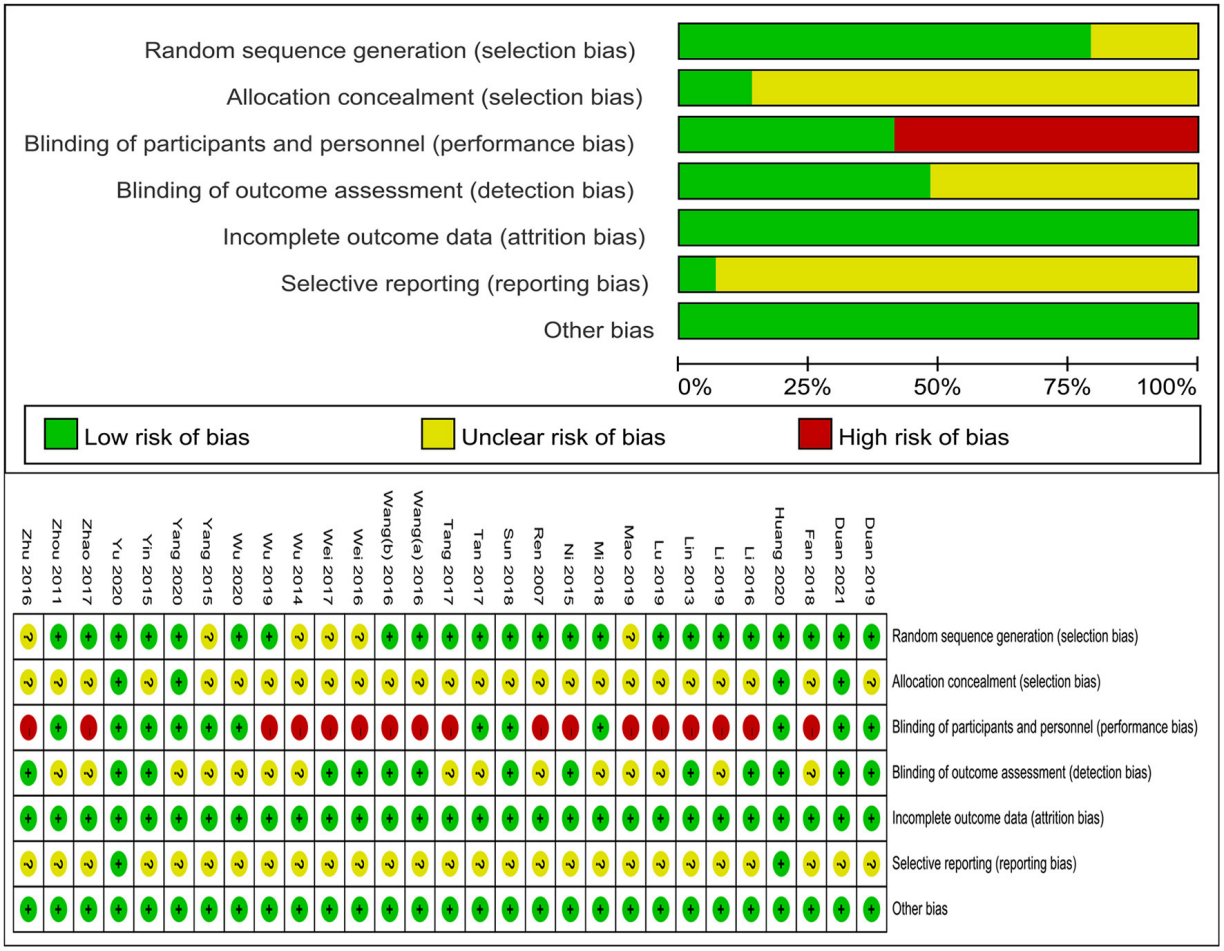
Risk of bias graph.

All included studies were described as randomized. Among them, 22 studies (27–29, 31, 32, 34, 37–39, 41–45, 48–55) (76%) were performed using a random number table, and 1 (47) adopted drawing lots. Four studies (51–53, 55) (13%) described the proper way to complete allocation concealment. Seventeen studies (27, 29–31, 34–40, 42, 43, 46–48, 50) (57%) were at high risk of bias for failing to blind participants and personnel, whereas the others were at low risk of bias. Fourteen studies (29, 31, 32, 34–38, 40, 45, 49, 51, 53, 55) (48%) were blinded to the outcome assessment. All studies (100%) showed low risks for incomplete outcome data. In this review, most of the research protocols (93%) were not available on record, and so selective reporting was difficult to judge. The methodological quality of the included trials is shown in Figure 2.

### Primary Outcomes

#### Incidence of POCD

**Figure 3:**
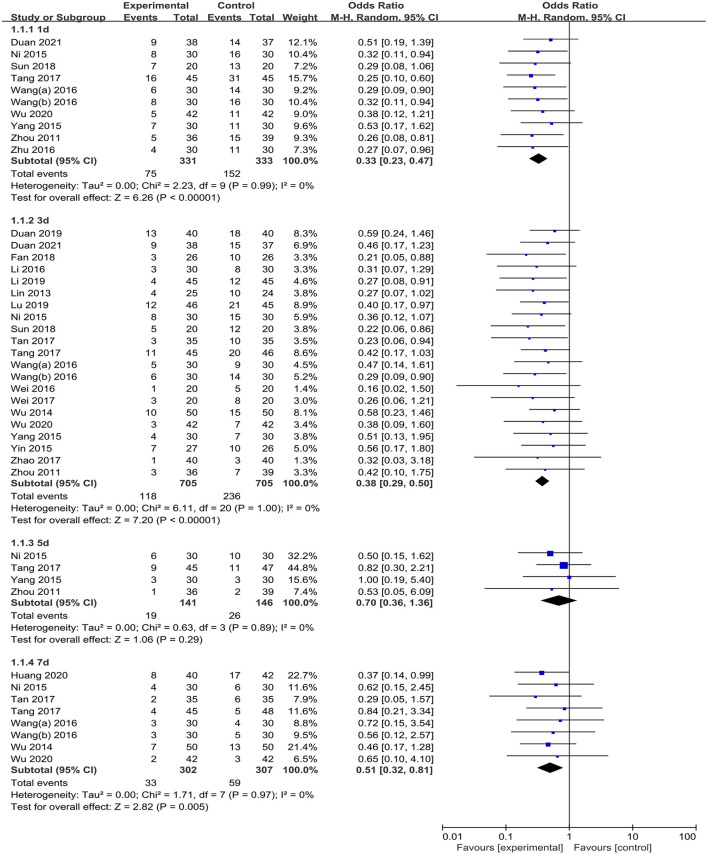
Meta-analysis and forest plot for the incidence of POCD at different periods.

Twenty-three studies (28–43, 45, 47, 49, 50, 53–55) reported the incidence of POCD. Overall, the results of the meta-analysis suggested a significantly lower incidence of POCD in the TEAS group than that in the CG [OR = 0.40 (95%CI: 0.33, 0.48); *p* < 0.001, *I*^2^ = 0%]. Subgroup analyses by evaluation time points (postoperative days 1, 3, 5, and 7) showed that the TEAS group had a significantly lower incidence of POCD compared with that in the CG on postoperative days 1 [OR = 0.33 (95%CI: 0.23, 0.47); *p* < 0.001, *I*^2^ = 0%, moderate certainty], 3 [OR = 0.38 (95%CI: 0.29, 0.50); *p* < 0.001, *I*^2^ = 0%, low certainty], and 7 [OR = 0.51 (95%CI: 0.32, 0.81); *p* = 0.005, *I*^2^ = 0%, low certainty]. On postoperative day 5, no significant difference was observed between the TEAS group and the CG for the incidence of POCD [OR = 0.70 (95%CI: 0.36, 1.36); *p* = 0.29, *I*^2^ = 0%, low certainty] (Figure 3 and Table 2). Subgroup analysis of the operation type showed that compared with the CG, TEAS significantly reduced the incidence of POCD in patients undergoing non-cardiac and noncraniocerebral surgery [OR = 0.40 (95%CI: 0.33, 0.49); *p* < 0.001, *I*^2^ = 0%, moderate certainty]. There was no significant difference in the incidence of POCD between the TEAS group and the CG, either for patients receiving craniocerebral surgery [only 1 study (39) reported, OR = 0.32 (95%CI: 0.03, 3.18); *p* = 0.33, *I*^2^ not applicable, low certainty] or cardiac surgery [only 1 study (53) reported, OR = 0.37 (95%CI: 0.14, 0.99); *p* = 0.05, *I*^2^ not applicable, moderate certainty]. Furthermore, a subgroup analysis based on the different anesthesia techniques suggested a significant reduction of POCD-related morbidity in the TEAS group, regardless of using intravenous anesthesia [OR = 0.44 (95%CI: 0.34, 0.56); *p* < 0.001, *I*^2^ = 0%, low certainty], intravenous-inhalation anesthesia [OR = 0.38 (95%CI: 0.27, 0.52); *p* < 0.001, *I*^2^ = 0%, low certainty], or combined spinal epidural anesthesia (CSEA) [OR = 0.35 (95%CI: 0.19, 0.62); *p* < 0.001, *I*^2^ = 0%, low certainty] (Supplementary Table 1).

**Table 2:** Main findings and evidence quality of the meta-analysis of TEAS for the prevention of POCD.

**Outcomes**	**No of studies (Participants)**	**Quality assessment**						**Effect (95% CI)**	**Quality**
		**Design**	**Risk of bias**	**Inconsistency**	**Indirectness**	**Imprecision**	**Other considerations**		
**Incidence of POCD**									
1d	10 studies (664)	RCT	Downgraded^a^	Not downgraded	Not downgraded	Not downgraded	Not downgraded	OR 0.33 (0.23, 0.47)	moderate
3d	21 studies (1410)	RCT	Downgraded^a^	Not downgraded	Not downgraded	Not downgraded	Downgraded^d^	OR 0.38 (0.29, 0.50)	low
5d	4 studies (287)	RCT	Downgraded^a^	Not downgraded	Not downgraded	Downgraded^c^	Not downgraded	OR 0.70 (0.36, 1.36)	low
7d	8 studies (609)	RCT	Downgraded^a^	Not downgraded	Not downgraded	Downgraded^c^	Not downgraded	OR 0.51 (0.32, 0.81)	low
**MMSE scores**									
1d	17 studies (1099)	RCT	Downgraded^a^	Downgraded^b^	Not downgraded	Not downgraded	Not downgraded	MD 2.44 (1.61, 3.27)	low
3d	17 studies (1096)	RCT	Downgraded^a^	Downgraded^b^	Not downgraded	Not downgraded	Not downgraded	MD 2.07 (1.53, 2.62)	low
5d	3 studies (210)	RCT	Downgraded^a^	Downgraded^b^	Not downgraded	Downgraded^c^	Not downgraded	MD 0.98 (−0.03, 1.99)	very low
7d	7 studies (506)	RCT	Downgraded^a^	Not downgraded	Not downgraded	Not downgraded	Downgraded^d^	MD 0.49 (0.18, 0.79)	low
**VAS scores**									
8 h	2 studies (175)	RCT	Downgraded^a^	Downgraded^b^	Not downgraded	Not downgraded	Not downgraded	MD−0.39 (−0.73, −0.05)	low
12 h	2 studies (164)	RCT	Downgraded^a^	Not downgraded	Not downgraded	Not downgraded	Not downgraded	MD−0.31 (−0.43, −0.20)	moderate
24 h	7 studies (510)	RCT	Downgraded^a^	Downgraded^b^	Not downgraded	Not downgraded	Not downgraded	MD−0.46 (−0.74, −0.17)	low
48 h	3 studies (226)	RCT	Downgraded^a^	Downgraded^b^	Not downgraded	Not downgraded	Not downgraded	MD−0.36 (−0.81, 0.10)	low
**Incidence of PONV**	9 studies (741)	RCT	Downgraded^a^	Not downgraded	Not downgraded	Downgraded^c^	Not downgraded	OR 0.36 (0.22, 0.58)	low
**Length of hospital stay**	1 study (52)	RCT	Downgraded^a^	Not downgraded	Not downgraded	Downgraded^c^	Not downgraded	MD−2.50 (−3.91, −1.09)	low

*TEAS, transcutaneous electrical acupoint stimulation; POCD, postoperative cognitive dysfunction; MMSE, mini-mental state examination; PONV, postoperative nausea and vomiting; VAS, visual analog scale; RCT, randomized controlled trial; CI, confidence intervals; OR, odds ratio; MD, mean difference*.

a *Downgraded by 1 level because unclear risk of bias was likely to lower confidence in the estimate of effect*.

b *Downgraded by 1 level because heterogeneity (I^2^ > 50%)*.

c *Downgraded by 1 level because total (cumulative) sample size was lower than the calculated optimal information size (OIS) and/or 95% CI included a null effect*.

d *Downgraded by 1 level because reporting bias (p < 0.1)*.

In the subgroup analysis of acupoint selection, we observed the efficacy of 6 kinds of acupoint combinations that were used more than 2 times in the included studies. Our metaanalysis showed that TEAS significantly reduced the incidence of POCD in 5 of the 6 combinations compared with CG. The acupoint combinations arranged in descending order of effect size were LI4, LI11, ST36, SP6 [OR = 0.33 (95%CI: 0.14, 0.77); *p* = 0.01, *I*^2^ = 0%, low certainty]; GV20, PC6, ST36, SP6 [OR = 0.37 (95%CI: 0.26, 0.54); *p* < 0.001, *I*^2^ = 0%, low certainty]; GV20, PC6, GB20 [OR = 0.38 (95%CI: 0.21, 0.71); *p* = 0.002, *I*^2^ = 0%, low certainty]; PC6, ST36 [OR = 0.40 (95%CI: 0.23, 0.69); *p* < 0.001, *I*^2^ = 0%, low certainty]; and LI4, PC6 [OR = 0.49 (95%CI: 0.31, 0.77); *p* = 0.002, *I*^2^ = 0%, low certainty]. Nevertheless, acupoint combination containing GV20, PC6, and ST36 in the TEAS group had no advantage in preventing POCD [OR = 0.53 (95%CI: 0.27, 1.04); *p* = 0.07, *I*^2^ = 0%, low certainty] (Supplementary Table 1).

The funnel plot of the above analysis showed no significant asymmetry (Supplementary Figure 1); moreover, the Harbord test showed no publication bias (*p* = 0.647; Supplementary Figures 2, 3). Additionally, the trim-and-fill method revealed that publication bias had little effect on the combined results and that the results were stable (Supplementary Figures 4, 5). Further sensitivity analysis by excluding low-quality studies showed that the pooled analysis results were stable (Supplementary Table 2).

#### Cognitive Function Score

**Figure 4:**
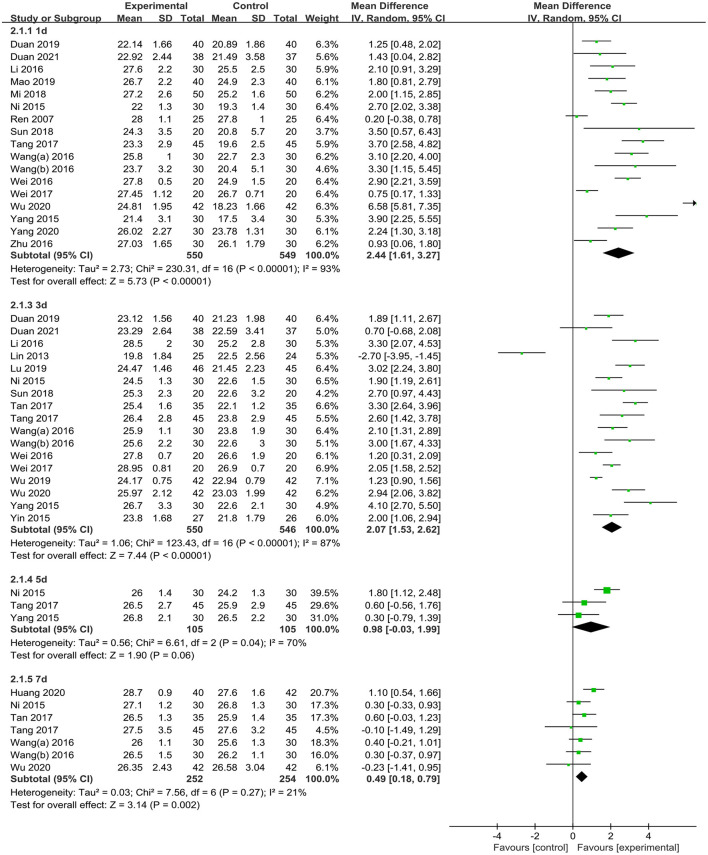
Meta-analysis and forest plot for MMSE scores at different periods.

The included articles applied 5 types of cognitive function scoring methods (including MMSE, MoCA, and QoR-15/40) used in the included articles. We eventually selected MMSE scores (*n* = 24 studies) to extract data for analysis (27, 29, 31–38, 40–42, 44–46, 48–55). One study (51) reported data in a figure, which was excluded from this analysis because of the absence of available data. The meta-analysis of different time points after surgery suggested that, compared with the CG, TEAS resulted in significantly improved MMSE scores on postoperative day 1 [MD = 2.44 (95%CI: 1.61, 3.27); *p* < 0.001, *I*^2^ = 93%, low certainty], 3 [MD = 2.07 (95%CI: 1.53, 2.62); *p* < 0.001, *I*^2^ = 87%, low certainty], and 7 [MD 0.49 = (95%CI: 0.18, 0.79); *p* = 0.002, *I*^2^ = 21%, low certainty] but not on day 5 [MD = 0.98 (95%CI: −0.03, 1.99); *P* = 0.06, *I*^2^ = 70%, very low certainty] (Figure 4 and Table 2).

Based on the above analysis, visual inspection showed an asymmetrical funnel plot (Supplementary Figure 6); however, Egger's test did not detect publication bias (*p* = 0.20; Supplementary Figures 7, 8). Therefore, we used the trim-and-fill method for further sensitivity analyses. After including the estimated missing studies, the imputed studies produced a symmetrical funnel plot, and the results after trimming and filling showed a significantly higher MMSE score in the TEAS group than that in the CG [MD = 1.48 (95%CI: 1.03, 1.93)] (Supplementary Figures 9, 10). Considering the substantial heterogeneity, further sensitivity analysis by excluding individual studies or low-quality studies suggested that the pooled analysis results were stable and no significant source of heterogeneity was found (Supplementary Table 2, Supplementary Figures 11–14).

### Secondary Outcomes

**Figure 5:**
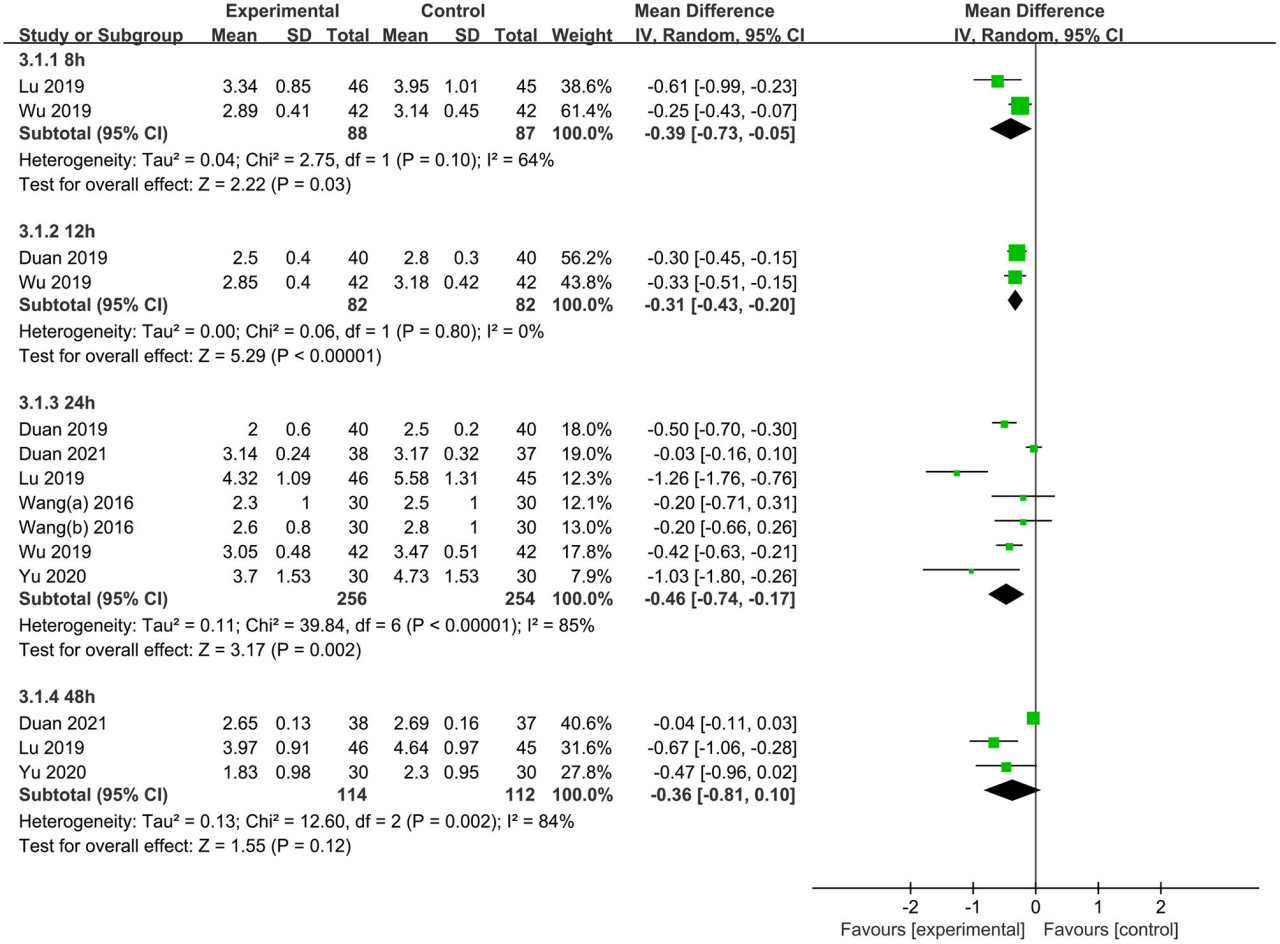
Meta-analysis and forest plot for VAS scores at different periods.

**Figure 6:**
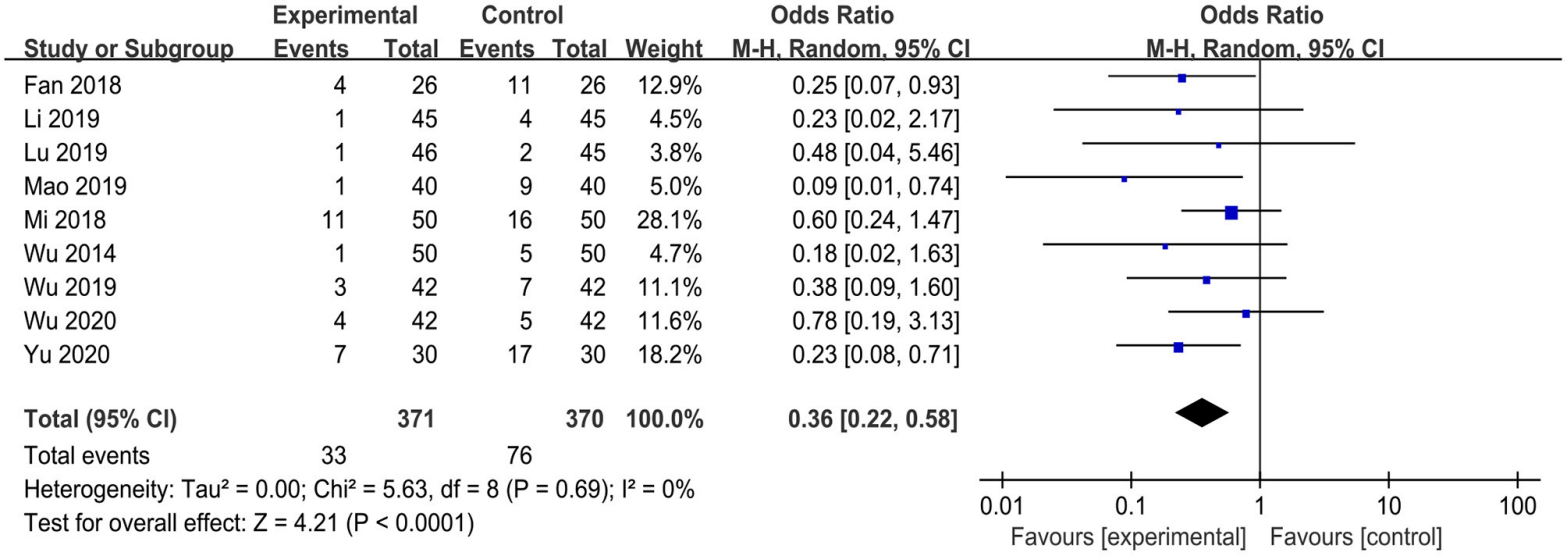
Meta-analysis and forest plot for the incidence of PONV.

**Figure 7:**
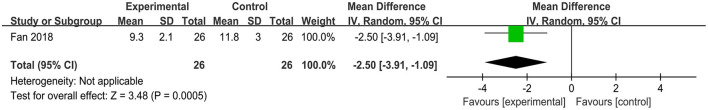
Meta-analysis and forest plot for the length of hospital stay.

Seven studies (34, 38, 48–51, 55) reported postoperative visual analog scale scores. The results of the meta-analysis demonstrated that TEAS had a significantly greater pain reduction compared with the CG at 8 h [MD = −0.39 (95%CI: −0.73, −0.05); *p* = 0.03, *I*^2^ = 64%, low certainty], 12 h [MD = −0.31 (95%CI: −0.43, −0.20); *p* < 0.001, *I*^2^ = 0%, moderate certainty], and 24 h [MD = −0.46 (95%CI: −0.74, −0.17); *p* = 0.002, *I*^2^ = 85%, low certainty] postoperatively, but not at 48 h [MD = −0.36 (95%CI: −0.81, 0.10); *p* = 0.12, *I*^2^ = 84%, low certainty] (Figure 5 and Table 2). Considering the significant heterogeneity between studies, we changed the pooled effect index to SMD. The heterogeneity was significantly reduced, and the pooled results remains statistically significant for 24 h after surgery (Supplementary Figure 15). Nine studies reported the incidence of PONV (30, 43, 44, 46–48, 50, 51, 54). Meta-analysis showed a significantly lower incidence of PONV in the TEAS group than in the CG [OR = 0.36 (95%CI: 0.22, 0.58); *p* < 0.001, *I*^2^ = 0%, low certainty] (Figure 6 and Table 2). Only 1 trial (43) was included in the meta-analysis of the effect of TEAS on the length of hospital stay. As shown in Figure 7 and Table 2, compared with the CG, the length of hospital stay in the TEAS group was significantly shorter [MD = −2.50 (95%CI: −3.91, −1.09); *p* < 0.001, low certainty].

## Discussion

Transcutaneous electrical acupoint stimulation has been widely used in clinical practice and has become an important part of perioperative management. TEAS provides many benefits in promoting rehabilitation after surgery from many aspects, including analgesia, gastrointestinal tract regulation, anti-inflammatory effects, stress response reduction, and POCD prevention (56). In this study, we examined the preventive effect of TEAS on postoperative short-term cognitive change. Our results showed that TEAS exerted a significant effect on increasing MMSE scores and lowering the incidence of POCD on postoperative days 1, 3, and 7. On a postoperative day 5, TEAS showed no significant therapeutic advantage in the primary outcomes, namely the incidence of POCD and MMSE scores. As reported in the literature, POCD, as a syndrome of prolonged impairment of cognitive function, occurs immediately to several years after surgery and usually persists for weeks or months (57). The effects of TEAS on medium- and long-term postoperative follow-up are still unclear. Anesthesia and surgery may be involved in the incidence of POCD. A previous meta-analysis showed that general anesthesia, compared to other forms, might increase the risk of developing POCD (58). A Cochrane systematic review suggested that, for elderly people undergoing non-cardiac surgery, maintenance with propofol-based total intravenous anesthesia might reduce the prevalence of POCD compared to maintenance with inhalational anesthesia (59). However, the effects of anesthesia and surgery on POCD remain controversial. A multicenter prospective cohort study indicated that exposure to surgery and general anesthesia were not significant risk factors for long-term cognitive impairment after major non-cardiac surgery associated with a critical illness (60). In this study, the subgroup analysis of anesthesia type showed that TEAS, compared with the CG, had a significant tendency toward a lower incidence of POCD, whether under general anesthesia (including intravenous and intravenous-inhalation) or non-general anesthesia. Furthermore, we found significantly reduced POCD in patients who underwent non-cardiac and noncraniocerebral surgery with TEAS. Of note, only one study involving cardiac surgery was included in this outcome. Based on the currently available data, there is insufficient evidence to recommend the use of TEAS in patients undergoing cardiac surgery.

The perioperative period is often accompanied by varying degrees of pain, which affects patient sleep, reduces the quality of life, and increases the length of hospital stay. TEAS do not only reduces pain and the incidence of complications caused by analgesics but also lessens the amount of anesthesia used in perioperative pain management (61). For postoperative pain, we found that TEAS enhanced the postoperative analgesic effect within 24 h after surgery. Furthermore, TEAS also effectively promoted the recovery of gastrointestinal function after surgery by increasing the level of plasma excitatory gastrointestinal hormone (62). A previous meta-analysis including 14 RCTs suggested that TEAS showed evidence of PONV prevention after general anesthesia (63), which was consistent with our findings. In general, POCD is associated with an increased length of hospital stay. Our review found that TEAS had advantages in shortening the postoperative hospital stay. Moreover, the selection of acupoints is an important factor in the TEAS treatment. The top 3 acupoints used in the included studies were PC6 (23 times), ST36 (15 times), and GV20 (14 times), which have the function of Kaiqiao Xingnao according to the theory of traditional Chinese medicine. As for the choice of acupoint combinations, we believe that the best combination is LI4, LI11, ST36, and SP6.

Transcutaneous electrical acupoint stimulation is “acupuncture-like TENS,” which is an ideal combination of acupoints and bioelectricity. Compared with traditional acupuncture or electroacupuncture, TEAS is a non-invasive therapy. The potential mechanism of TEAS in preventing POCD may be explained by the following: first, TEAS alleviates postoperative inflammatory injury and reduces cytokine interleukin-1β (IL-1β) and tumor necrosis factor-α (TNF-α) levels in the central nervous system and peripheral circulation (32, 49, 53, 64); second, TEAS can reduce hippocampal neurons apoptosis by increasing the Bcl-2/Bax ratio and inhibiting activated caspase-3 expression (64); third, TEAS regulates serum neuron-specific enolase and S100-β protein (S100-β) levels (28, 36), or reduces the oxidative stress reaction (31); fourth, TEAS enhances the effect of postoperative analgesia and reduces the dosage of analgesics (65).

A recent expert consensus concluded that prevention was the best treatment for postoperative cognitive impairment and proposed some practical recommendations, such as pain control, cognitive screening, and minimizing psychoactive treatments (12, 66, 67). However, the recommended brain assessments and simple preventive measures were not routinely implemented and need further work to identify potential strategies with high therapy compliance (68). As a new type of acupuncture therapy, TEAS has attracted the attention of many researchers because of its multiple advantages, such as unified parameters, simple operation, and good patient compliance. The results of our review provided low to moderate certainty of evidence that TEAS was beneficial for the prevention of POCD in patients undergoing anesthesia and surgery, and it significantly reduced the incidence of PONV. We also observed the benefits of TEAS in improving neurological function scores, although the evidence level was downgraded to low due to statistical heterogeneity. Low to moderate certainties of evidence suggested that TEAS could enhance postoperative analgesia and shorten hospital stay. In general, these findings may provide new strategies for the management of perioperative brain health guidelines in the future.

## Limitations

This review has several limitations. First, the long-term follow-up results are unknown because the observation time of the included studies was generally within 7 days after surgery. Second, all 29 included studies were conducted in China and most had small sample sizes (≤ 100), which may have affected the reliability and extrapolation of the overall results. Due to the small sample size of the included studies, the estimated results were likely overestimated. Consequently, we performed a subgroup analysis to explore the impact of small sample sizes by grouping their sample size into quarters. The forest plot showed that the overall pooled results were not overestimated (*p* > 0.05) (Supplementary Figure 16). Generally speaking, the meta-analysis included no trials with large sample sizes; thus, the detection strength was also limited. Third, some of the studies had high risk and low quality, which downregulated the evidence strength of the research results. Therefore, future trials with higher-quality and larger sample sizes are needed to make more firm conclusions.

## Conclusion

Our review systematically investigated and quantified the preventive effects of TEAS on POCD. Overall, our findings suggested that the application of TEAS in the perioperative period was associated with improved cognitive function scores and reduced POCD rates in the early postoperative period. Meanwhile, limited evidence suggested that TEAS could enhance the postoperative analgesic effect within 24 h after surgery, decrease PONV incidence, and reduce the length of hospital stay. Large RCTs are needed to determine the preventive effects of TEAS for POCD before recommending its routine use in surgical practice.

## Data Availability Statement

The original contributions presented in the study are included in the article/Supplementary Material, further inquiries can be directed to the corresponding author/s.

## Author Contributions

GH conceived the original study design. TZ and LO developed the search strategy and searched the literature database. JL and YS screened the eligible studies and extracted the data. JL and TZ evaluated the risk of bias. LO and ZC performed data analysis and evaluated the certainty of evidence. TZ drafted the first manuscript and GH revised the manuscript. All authors read and approved the submitted version.

## Funding

This study was supported by the National Natural Science Foundation of China (no. 81573941); Hunan Natural Science Foundation general project (no. 2020JJ4476); the Hunan University of Chinese Medicine, domestic first-class construction discipline, Open Fund Project of First-class Discipline of Hunan University of Chinese Medicine (no. 2021ZYX40); Hu Guoheng Famous Doctor Inheritance Studio.

## Conflict of Interest

The authors declare that the research was conducted in the absence of any commercial or financial relationships that could be construed as a potential conflict of interest.

## Publisher's Note

All claims expressed in this article are solely those of the authors and do not necessarily represent those of their affiliated organizations, or those of the publisher, the editors and the reviewers. Any product that may be evaluated in this article, or claim that may be made by its manufacturer, is not guaranteed or endorsed by the publisher.
