# Supplementary material for: Transcutaneous Electrical Acupoint Stimulation for the Prevention of Postoperative Cognitive Dysfunction: A Systematic Review and Meta-Analysis
Source: Front Med (Lausanne). 2021 Dec 6;8:756366. doi: 10.3389/fmed.2021.756366 (PMC8685241; doi:10.3389/fmed.2021.756366)
Supplement: Supplementary file 1 [file Data_Sheet_1.docx]

Supplementary Material

**The search strategy used for EMBASE**

#1 'postoperative cognitive dysfunction'/exp 1,391

#2 'postoperative cognitive complications':ab,ti 14

#3 'postoperative cognitive complication':ab,ti 0

#4 'postoperative decline':ab,ti 291

#5 'postoperative declines':ab,ti 26

#6 'cognitive dysfunction':ab,ti 22,129

#7 'cognitive dysfunctions':ab,ti 2,327

#8 'cognitive impairments':ab,ti 13,859

#9 'cognitive impairment':ab,ti 97,755

#10 'neurocognitive disorder':ab,ti 1,543

#11 'neurocognitive disorders':ab,ti 3,214

#12 'cognitive decline':ab,ti 37,837

#13 'cognitive declines':ab,ti 503

#14 'mental deterioration':ab,ti 1,486

#15 'mental deteriorations':ab,ti 2

#16 'cognition':ab,ti 102,992

#17 'cognitions':ab,ti 8,026

#18 'cognitive function':ab,ti 55,832

#19 'cognitive functions':ab,ti 26,884

#20 #1 OR #2 OR #3 OR #4 OR #5 OR #6 OR #7 OR #8 OR #9 OR #10 OR #11 OR #12 OR #13 OR #14 OR #15 OR #16 OR #17 OR #18 OR #19 286,040

#21 'electrostimulation'/exp 86,532

#22 'acupuncture point'/exp 2,393

#23 'acupuncture point':ab,ti 926

#24 'acupuncture points':ab,ti 2,081

#25 'acupoints':ab,ti 4,307

#26 'acupoint':ab,ti 3,764

#27 'electric stimulation':ab,ti 4,942

#28 'electric stimulations':ab,ti 135

#29 'electrostimulation':ab,ti 4,085

#30 'electrostimulations':ab,ti 46

#31 #21 OR #27 OR #28 OR #29 OR #30 90,711

#32 #22 OR #23 OR #24 OR #25 OR #26 10,025

#33 #31 AND #32 558

#34 'transcutaneous acupoint electrical stimulation':ab,ti 34

#35 'transcutanclus electrical acupoint stimulation':ab,ti 3

#36 'transcutaneous point electric stimulation':ab,ti 0

#37 'percutaneous acupoint electrical stimulation':ab,ti 0

#38 'electrical acupoint stimulation':ab,ti 167

#39 'transcutaneous electrical acupoint stimulation':ab,ti 152

#40 #34 OR #35 OR #36 OR #37 OR #38 OR #39 200

#41 #33 OR #40 724

#42 #20 AND #41 7

**The search strategy used for PubMed**

#1 "Cognitive Dysfunction"[Mesh] 21,485

#2 (((((((((((((Cognitive Dysfunction[Title/Abstract]) OR (Cognitive Dysfunctions[Title/Abstract])) OR (Cognitive Impairments[Title/Abstract])) OR (Cognitive Impairment[Title/Abstract])) OR (Neurocognitive Disorder[Title/Abstract])) OR (Neurocognitive Disorders[Title/Abstract])) OR (Cognitive Decline[Title/Abstract])) OR (Cognitive Declines[Title/Abstract])) OR (Mental Deterioration[Title/Abstract])) OR (Mental Deteriorations[Title/Abstract])) OR (Cognitions[Title/Abstract])) OR (Cognition[Title/Abstract])) OR (Cognitive Function[Title/Abstract])) OR (Cognitive Functions[Title/Abstract]) 207565

#3 "Postoperative Cognitive Complications"[Mesh] 184

#4 (((Postoperative Cognitive Complications[Title/Abstract]) OR (Postoperative Cognitive Complication[Title/Abstract])) OR (Postoperative Decline[Title/Abstract])) OR (Postoperative Declines[Title/Abstract]) 235

#5 "Acupuncture Points"[Mesh] 7037

#6 (((Acupuncture Points[Title/Abstract]) OR (Acupuncture Point[Title/Abstract])) OR (Acupoints[Title/Abstract])) OR (Acupoint[Title/Abstract]) 7245

#7 "Electric Stimulation"[Mesh] 127453

#8 (((Electric Stimulation[Title/Abstract]) OR (Electrical Stimulation[Title/Abstract])) OR (Electrical Stimulations[Title/Abstract])) OR (Electric Stimulations[Title/Abstract]) 52274

#9 #1 OR #2 OR #3 OR #4 210584

#10 #5 OR #6 10508

#11 #7 OR #8 152009

#12 #10 AND #11 563

#13 (((((transcutaneous acupoint electrical stimulation[Title/Abstract]) OR (Transcutaneous Electrical Acupoint Stimulation[Title/Abstract])) OR (Transcutaneous point electric stimulation[Title/Abstract])) OR (Percutaneous acupoint electrical stimulation[Title/Abstract])) OR (transcutanclus electrical acupoint stimulation[Title/Abstract])) OR (Electrical Acupoint Stimulation[Title/Abstract]) 200

#14 #12OR #13 688

#15 #9 AND #14 9

**The search strategy used for CENTRAL**

#1 MeSH descriptor: [Cognitive Dysfunction] explode all trees 1675

#2 (Cognitive Dysfunction):ti,ab,kw OR (Cognitive Dysfunctions):ti,ab,kw OR (Cognitive Impairments):ti,ab,kw OR (Cognitive Impairment):ti,ab,kw OR (Neurocognitive Disorder):ti,ab,kw (Word variations have been searched) 22682

#3 (Neurocognitive Disorders):ti,ab,kw OR (Cognitive Decline):ti,ab,kw OR (Cognitive Declines):ti,ab,kw OR (Mental Deterioration):ti,ab,kw OR (Mental Deteriorations):ti,ab,kw (Word variations have been searched) 8599

#4 (Cognition):ti,ab,kw OR (Cognitions):ti,ab,kw OR (Cognitive Function):ti,ab,kw OR (Cognitive Functions):ti,ab,kw (Word variations have been searched) 80398

#5 MeSH descriptor: [Postoperative Cognitive Complications] explode all trees 19

#6 (Postoperative Cognitive Complications):ti,ab,kw OR (Postoperative Cognitive Complication):ti,ab,kw OR (Postoperative Decline):ti,ab,kw OR (Postoperative Declines):ti,ab,kw (Word variations have been searched) 2317

#7 #1 OR #2 OR #3 OR #4 OR #5 OR #6 82667

#8 MeSH descriptor: [Acupuncture Points] explode all trees 2054

#9 MeSH descriptor: [Electric Stimulation] explode all trees 1911

#10 (Acupuncture Point):ti,ab,kw OR (Acupuncture Points):ti,ab,kw OR (Acupoints):ti,ab,kw OR (Acupoint):ti,ab,kw (Word variations have been searched) 7951

#11 (Electric Stimulation):ti,ab,kw OR (Electric Stimulations):ti,ab,kw OR (Electrical Stimulation):ti,ab,kw OR (Electrical Stimulations):ti,ab,kw (Word variations have been searched)

12623

#12 #8 OR #10 7951

#13 #9 OR #11 12750

#14 #12 AND #13 962

#15 (transcutaneous acupoint electrical stimulation):ti,ab,kw OR (Transcutaneous Electrical Acupoint Stimulation):ti,ab,kw OR (Transcutaneous point electric stimulation):ti,ab,kw OR (Percutaneous acupoint electrical stimulation):ti,ab,kw AND (transcutanclus electrical acupoint stimulation):ti,ab,kw (Word variations have been searched) 887

#16 #14 OR #15 1276

#17 #7 AND#16 43

**The search strategy used for CBM**

#1 ("经皮神经电刺激"[不加权:扩展]) AND "穴位疗法"[不加权:扩展] 1918

#2 穴位电刺激 1026

#3 穴位 AND 电刺激 2050

#4 #1 OR #2 OR #3 3915

#5 "认知障碍"[不加权:扩展] 19403

#7 认知 161757

#9 POCD 2261

#10 #7 OR #6 OR #5 162528

#11 #8 AND #4 72

**The search strategy used for CNKI (n = 64) and Wan Fang Database (n = 135)**

(主题: 穴位电刺激) OR (主题：穴位 * 电刺激) AND ( (主题: 认知 + POCD) )

**The search strategy used for VIP**

(题名或关键词: 穴位 * 电刺激) + (题名或关键词: 穴位电刺激) AND (题名或关键词: 认知 + POCD) 41

Supplementary File 1. The detailed search strategy.

**Supplementary Table 1** Subgroup analysis for the incidence of POCD.

| **Outcomes** | **No. of Studies** | **No. of Participants** | **Effect Estimate**  **(95% CI)** | ***I^2^***  **Heterogeneity, %** | **GRADE** |
| --- | --- | --- | --- | --- | --- |
| **Type of operation** |  |  |  |  |  |
| Craniocerebral surgery | 1 | 80 | 0.32 (0.03, 3.18) | NA | low |
| Cardiac surgery | 1 | 82 | 0.37 (0.14, 0.99) | NA | moderate |
| Non cardiac or craniocerebral surgery | 21 | 2808 | 0.40(0.33, 0.49) | 0 | moderate |
| **Type of anesthesia** |  |  |  |  |  |
| Intravenous | 9 | 1552 | 0.44 (0.34, 0.56) | 0 | low |
| Intravenous-inhalation | 11 | 1018 | 0.38 (0.27, 0.52) | 0 | low |
| CSEA | 2 | 260 | 0.35 (0.19, 0.62) | 0 | low |
| **Acupoint combination** |  |  |  |  |  |
| LI4, LI11, ST36, SP6 | 1 | 225 | 0.33 (0.14, 0.77) | 0 | low |
| GV20, PC6, ST36 | 1 | 200 | 0.53 (0.27, 1.04) | 0 | low |
| GV20, PC6, ST36, SP6 | 4 | 740 | 0.37 (0.26, 0.54) | 0 | low |
| GV20, PC6, GB20 | 4 | 224 | 0.38 (0.21, 0.71) | 0 | low |
| PC6, ST36 | 4 | 390 | 0.40 (0.23, 0.69) | 0 | low |
| LI4, PC6 | 3 | 482 | 0.49 (0.31, 0.77) | 0 | low |

CSEA, combined spinal epidural anesthesia; CI, confidence intervals; GRADE, The Grading of Recommendations Assessment, Development and Evaluation; NA, not available.

**Supplementary Table 2** Sensitivity analysis by eliminating articles with poor quality.

| **Outcomes** | **No. of Studies** | **No. of Participants** | **Effect Estimate**  **(95% CI)** | ***I^2^* Heterogeneity, %** |
| --- | --- | --- | --- | --- |
| incidence of POCD |  |  |  |  |
| 1d | 5 | 334 | 0.39 (0.23, 0.65) | 0 |
| 3d | 8 | 537 | 0.43 (0.28, 0.65) | 0 |
| 5d | 2 | 135 | 0.81 (0.20, 3.26) | 0 |
| 7d | 3 | 236 | 0.39 (0.18, 0.84) | 0 |
| MMSE scores |  |  |  |  |
| 1d | 7 | 499 | 2.97 (1.20, 4.73) | 95 |
| 3d | 7 | 462 | 2.53 (1.81, 3.25) | 72 |
| 5d | 1 | 60 | 0.30 (-0.79, 1.39) | NA |
| 7d | 3 | 236 | 0.65 (0.02, 1.28) | 54 |

CI, confidence intervals; POCD, postoperative cognitive dysfunction; MMSE, mini-mental state examination; NA, not available.

**
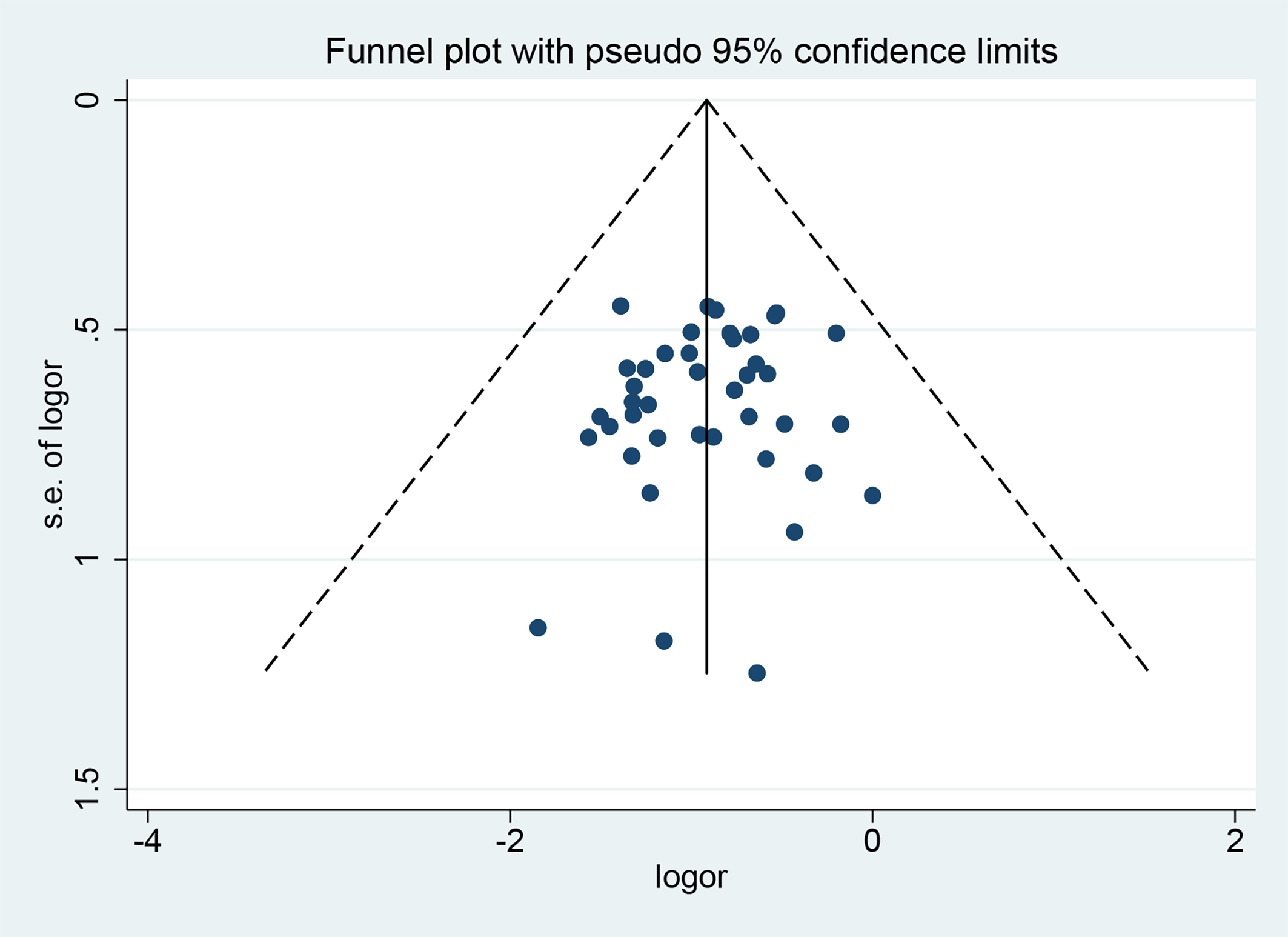
**

Supplementary Figure 1. Funnel plot for the incidence of POCD.


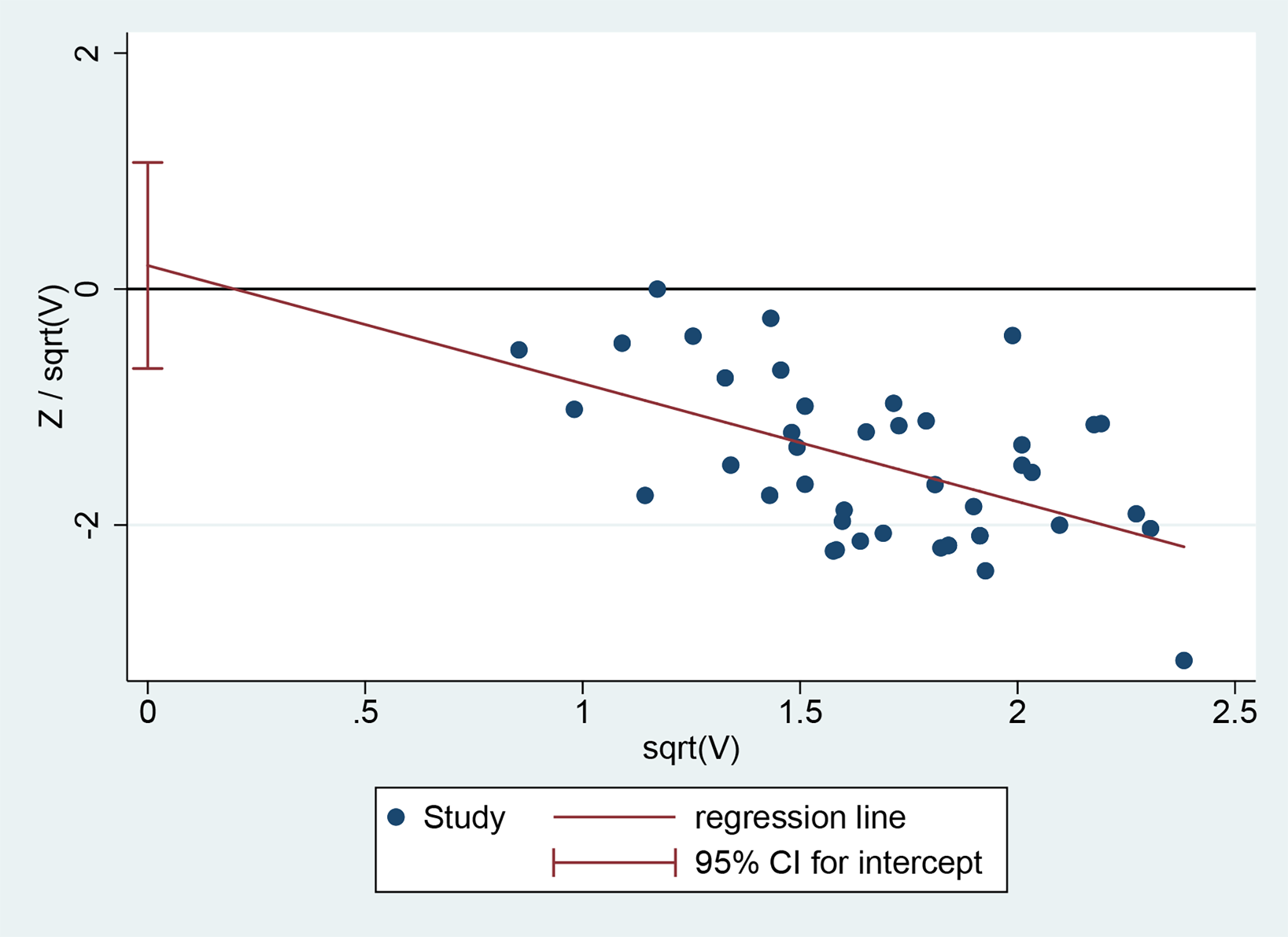


**Supplementary Figure 2.** Regression plot of Harbord test for the incidence of POCD.


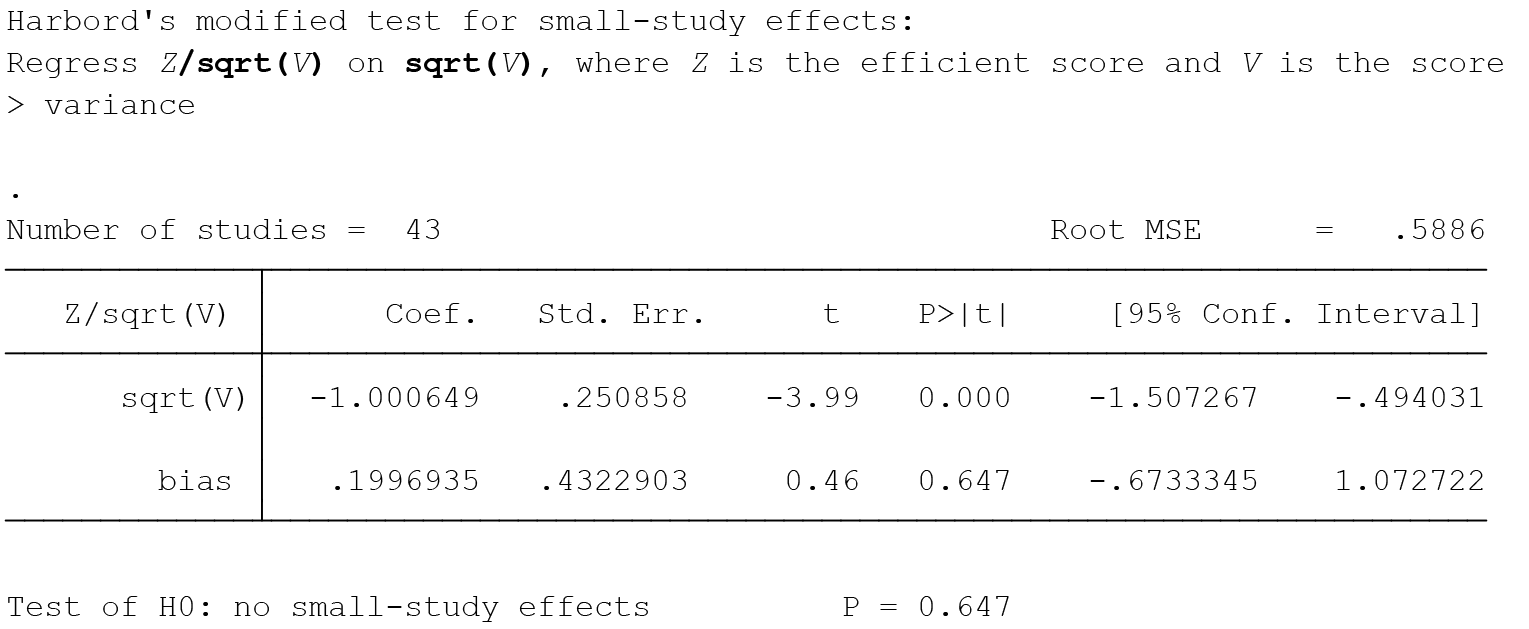


**Supplementary Figure 3.** Harbord test for the incidence of POCD.


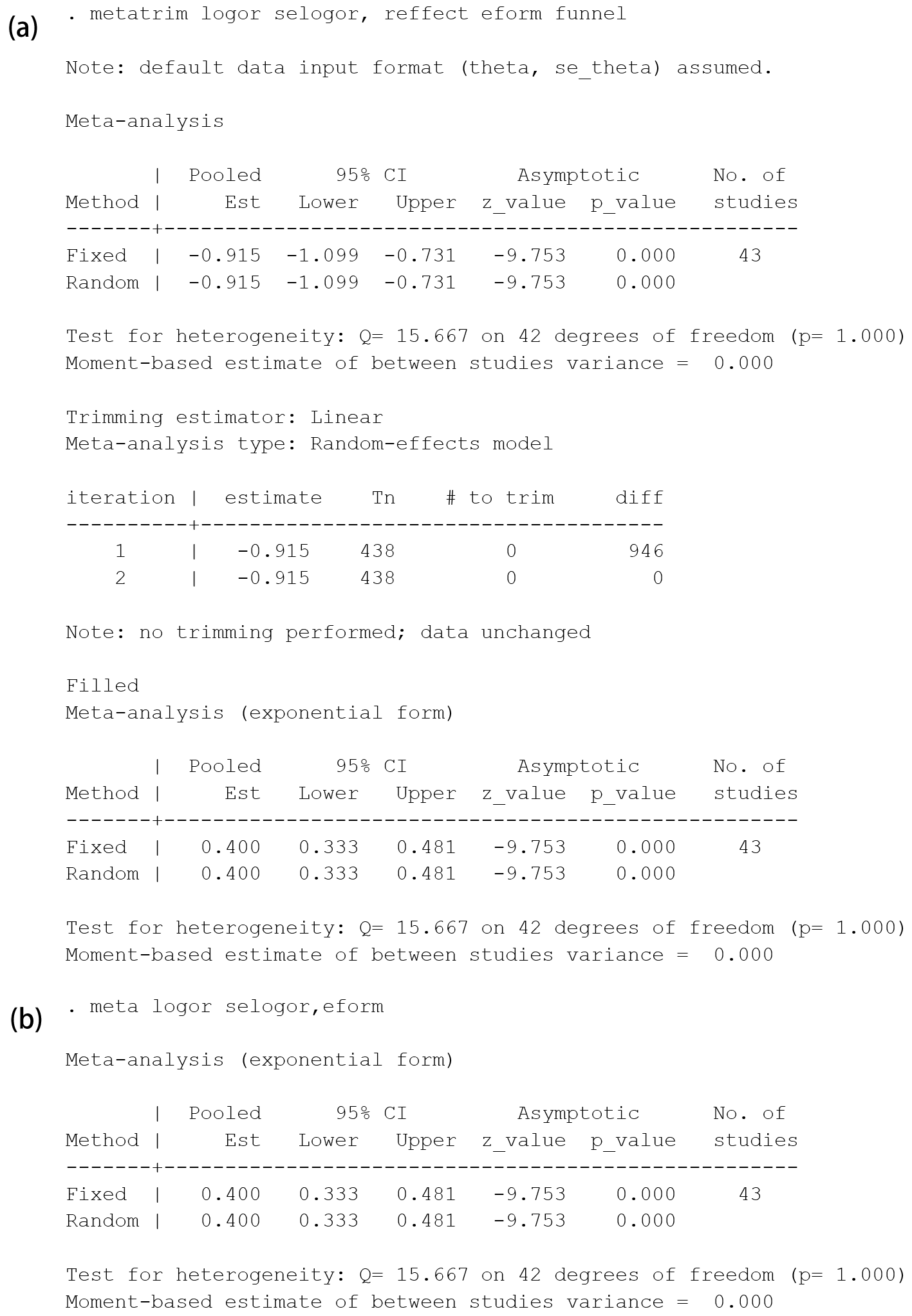


**Supplementary Figure 4.** Trim-and-fill method for the incidence of POCD. **(a)** The log OR of fixed effect model and random effect model before trimming and filling was same [log OR = -0.92 (95%CI: -1.10, -0.73)], and trim-and-fill method did not reduce or increase studies. **(b)** The pooled log OR before trimming and filling was converted to OR. The results didn’t change before or after trimming and filling [OR = 0.40 (95%CI: 0.33, 0.48)].


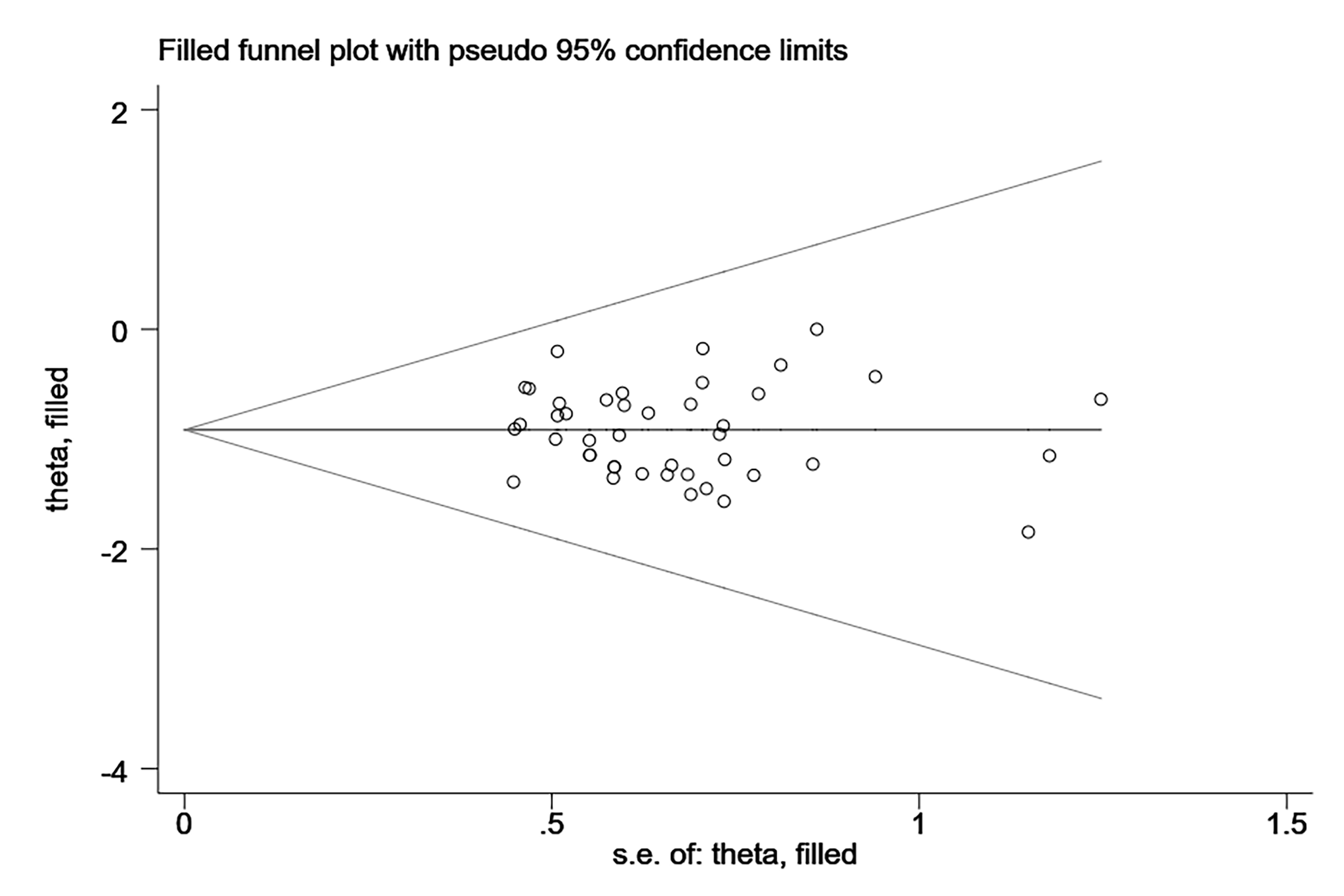


**Supplementary Figure 5.** Funnel plot for the incidence of POCD after trimming and filling.


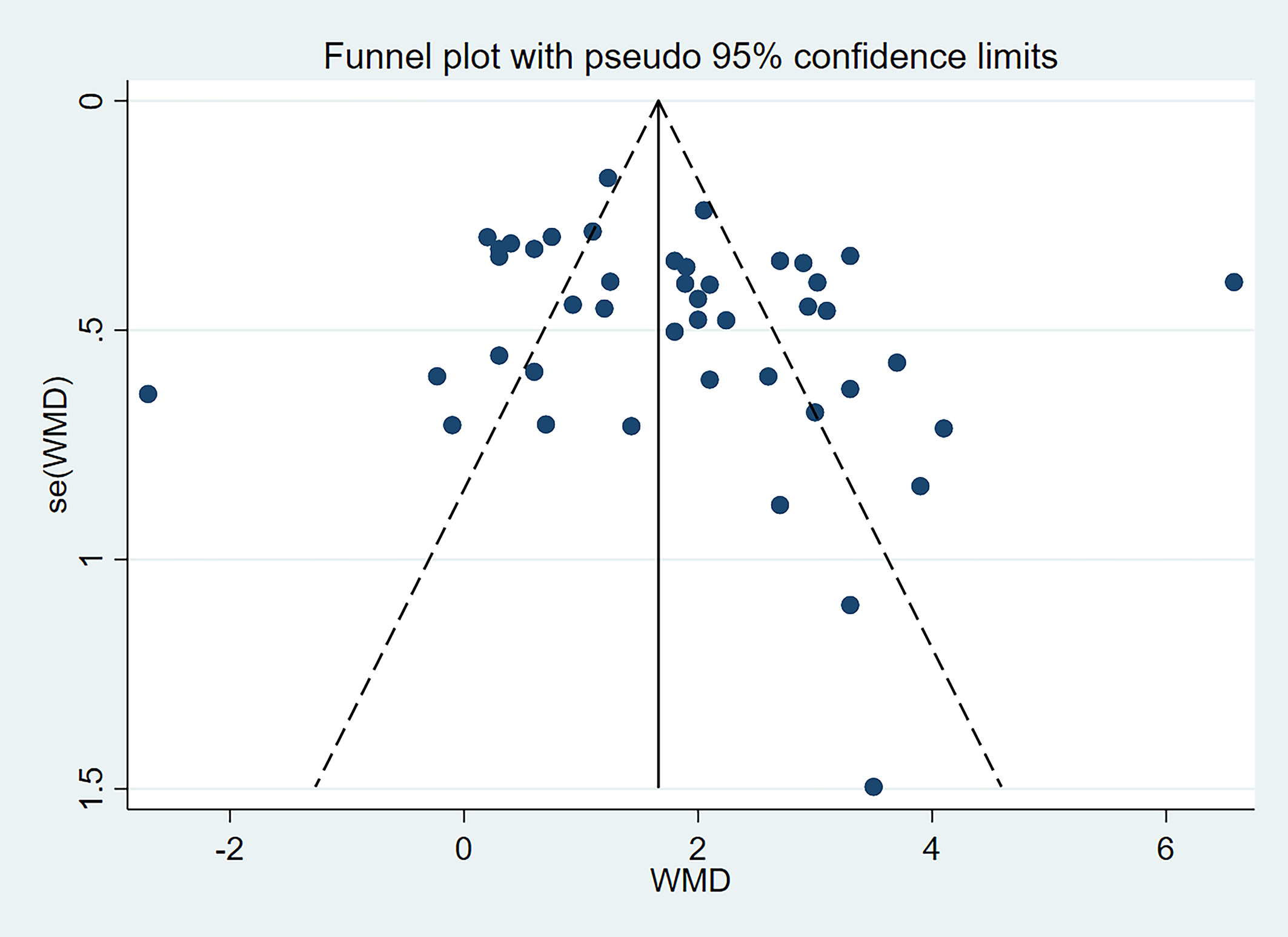


**Supplementary Figure 6.** Funnel plot for the MMSE scores.


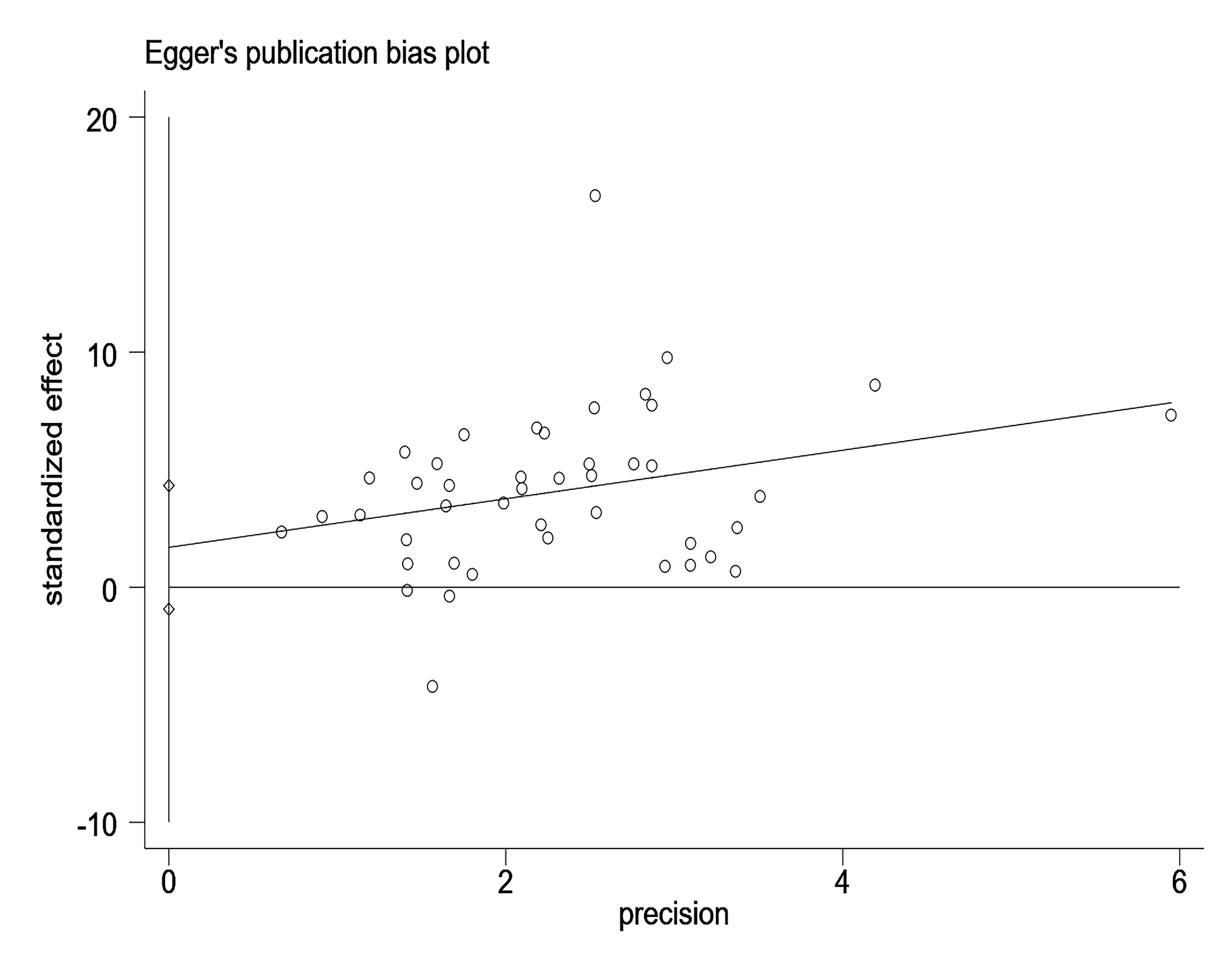


**Supplementary Figure 7.** Regression plot of Egger's test for the MMSE scores.


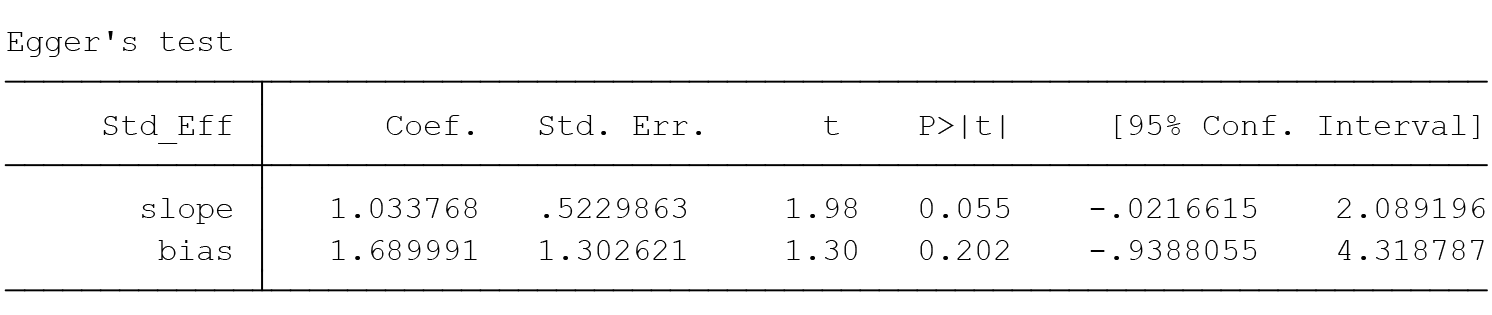


**Supplementary Figure 8.** Egger's test for the MMSE scores.

.


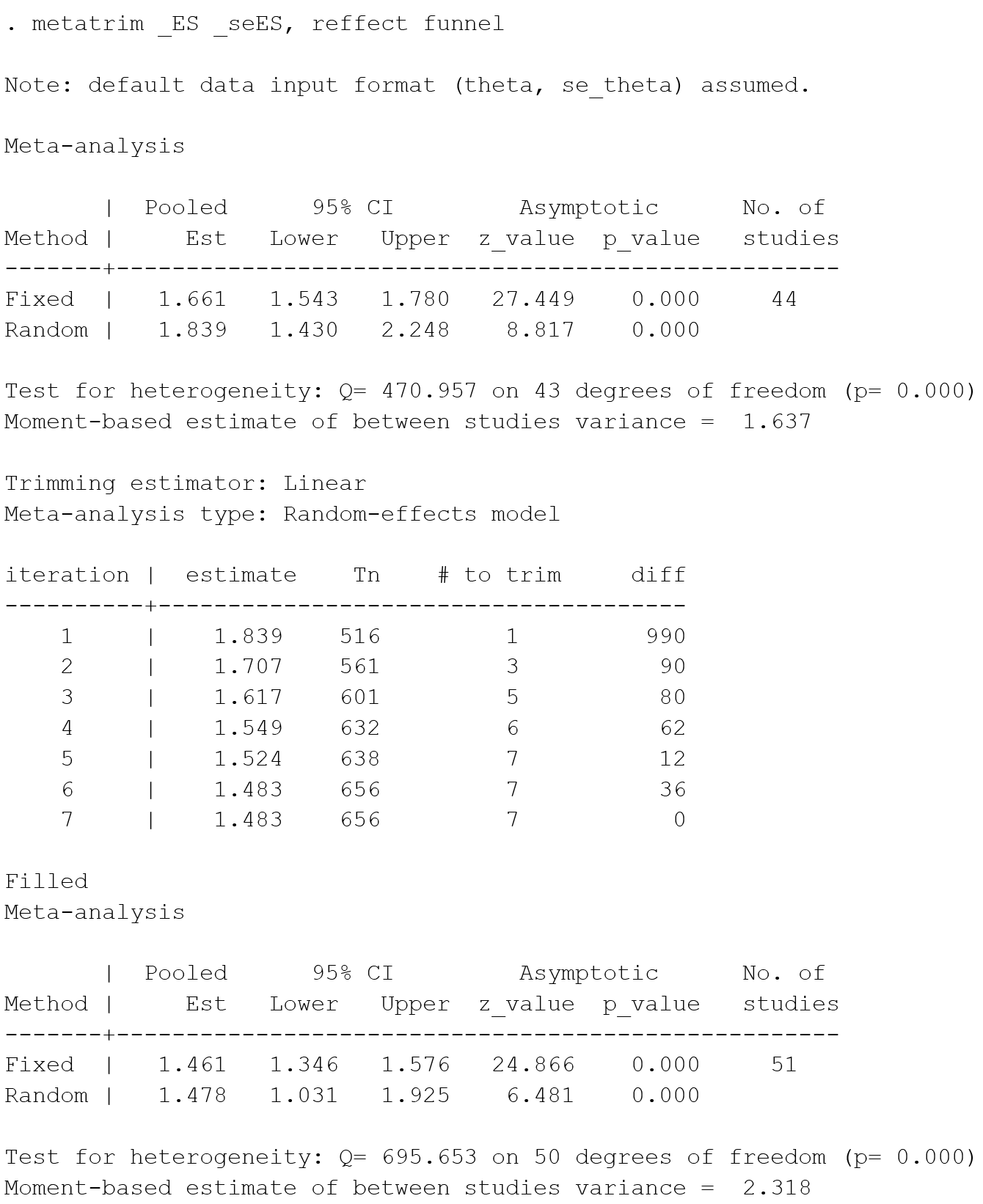


**Supplementary Figure 9.** Trim-and-fill method for the MMSE scores. Before trimming and filling, the pooled MD of the fixed effect model was 1.66 with 95% CI (1.54, 1.78) and of the random-effects model was 1.84 with 95% CI (1.43, 2.25). The number of missing studies was estimated using the random effects model and linear method after 7 iterations (finally diff = 0), resulting in 7. Then, after including the estimated missing studies, the pooled MD from the fixed-effects model was 1.46 with 95% CI (1.35, 1.58), and from the random-effects model was 1.48 with 95% CI (1.03, 1.93).


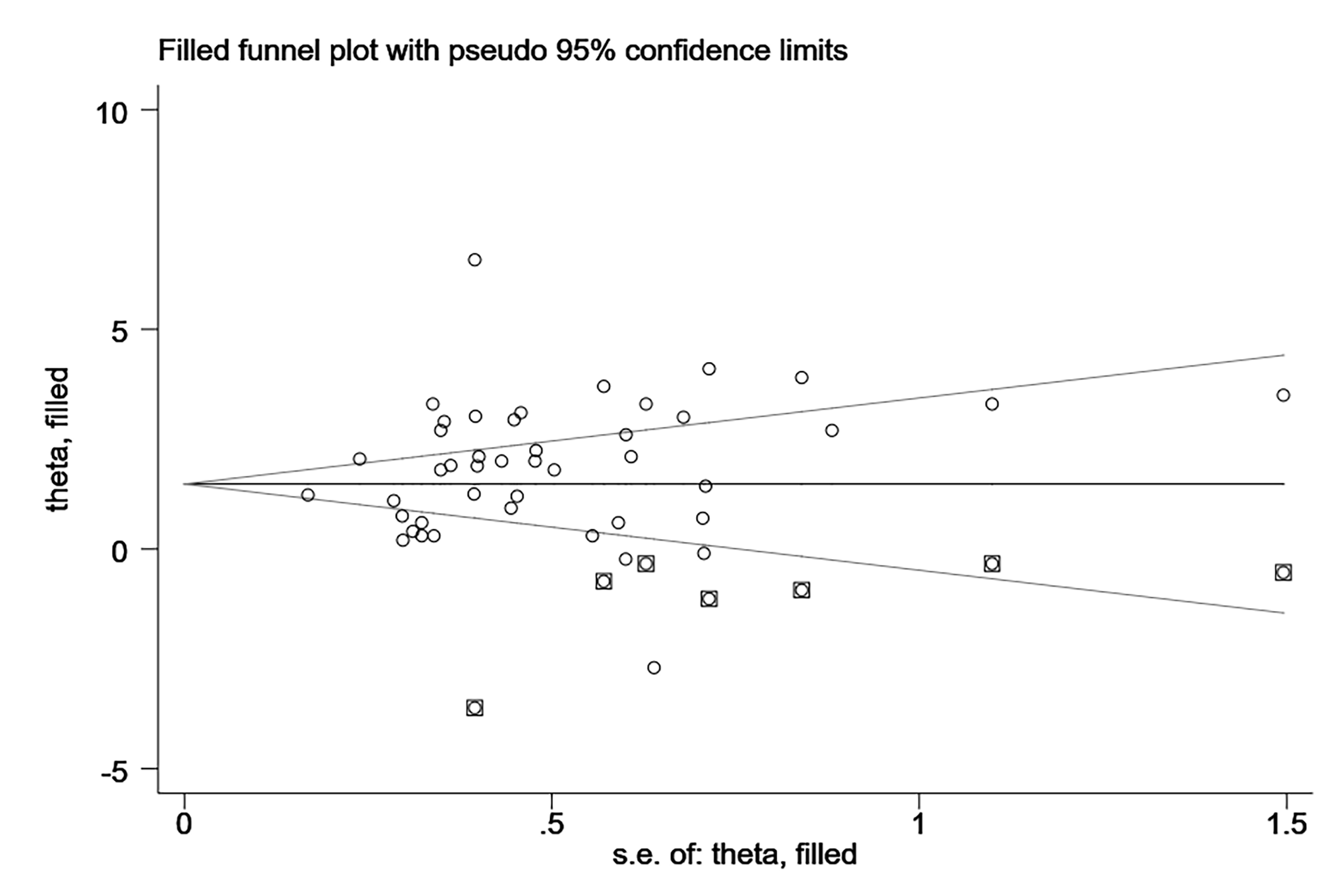


**Supplementary Figure 10.** Funnel plot for the MMSE scores after trimming and filling. Seven missing studies shown as “squares” in the figure were filled in the plot.


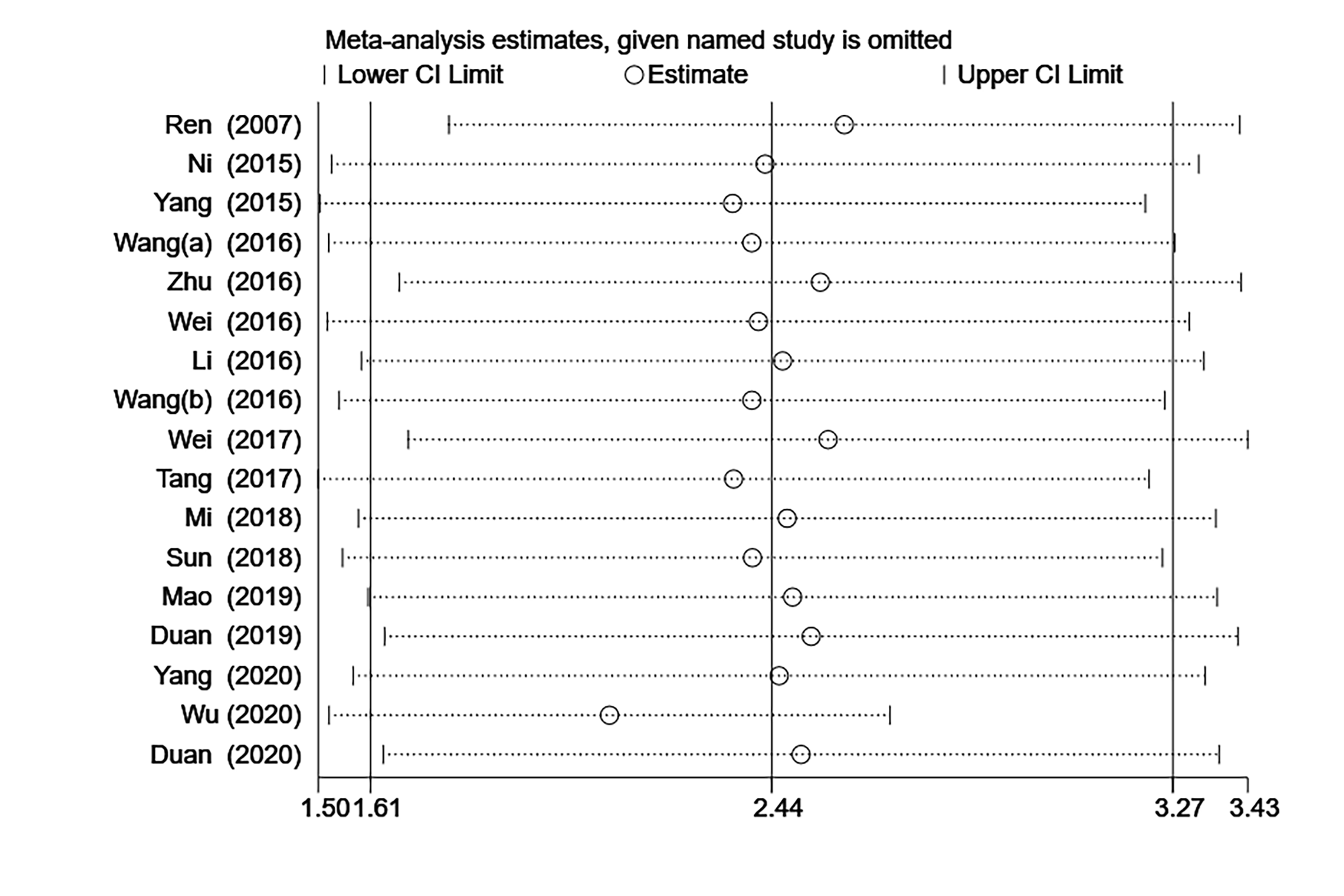


**Supplementary Figure 11.** Sensitivity analysis for the MMSE scores on post-operative 1d.


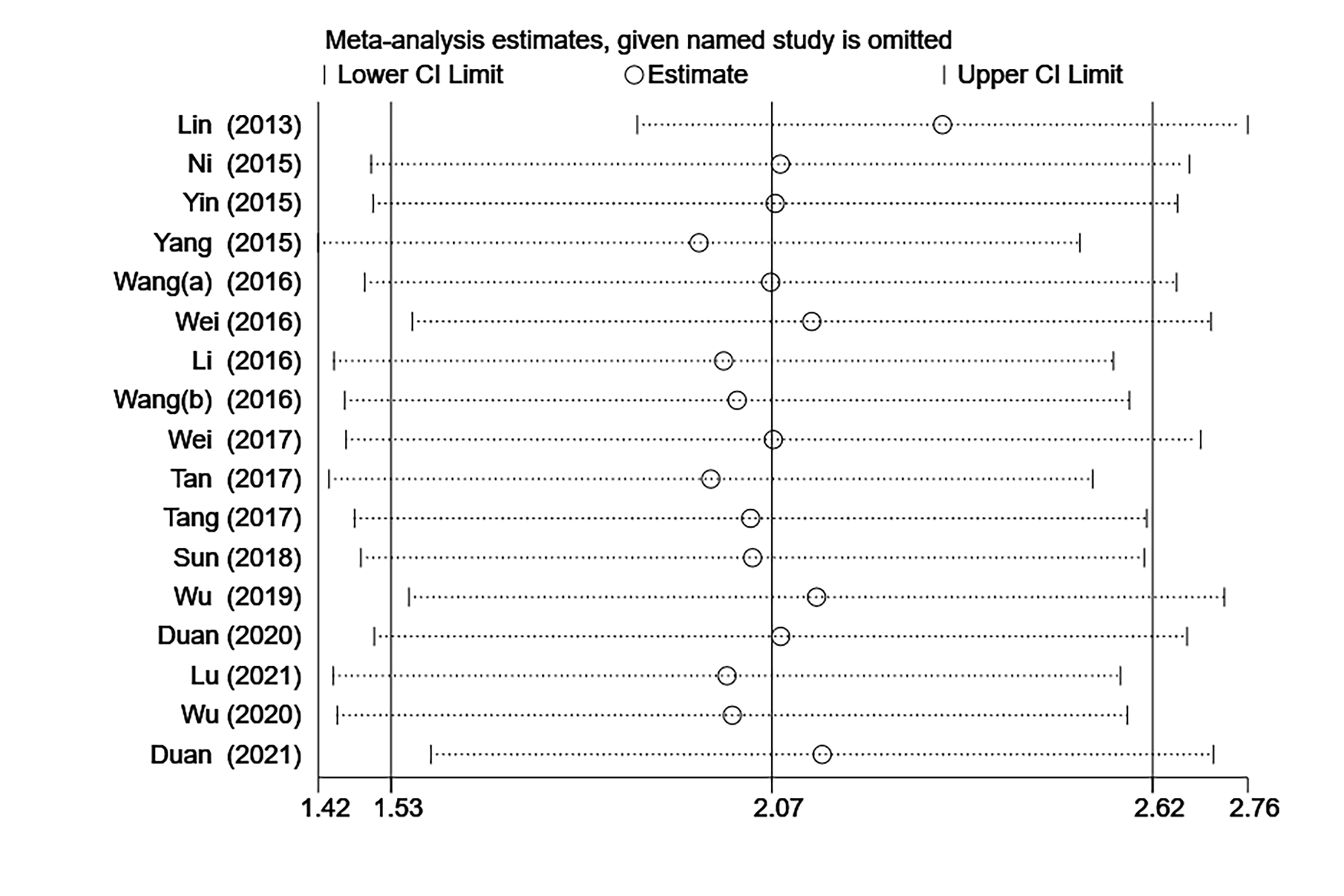


**Supplementary Figure 12.** Sensitivity analysis for the MMSE scores on post-operative 3d.


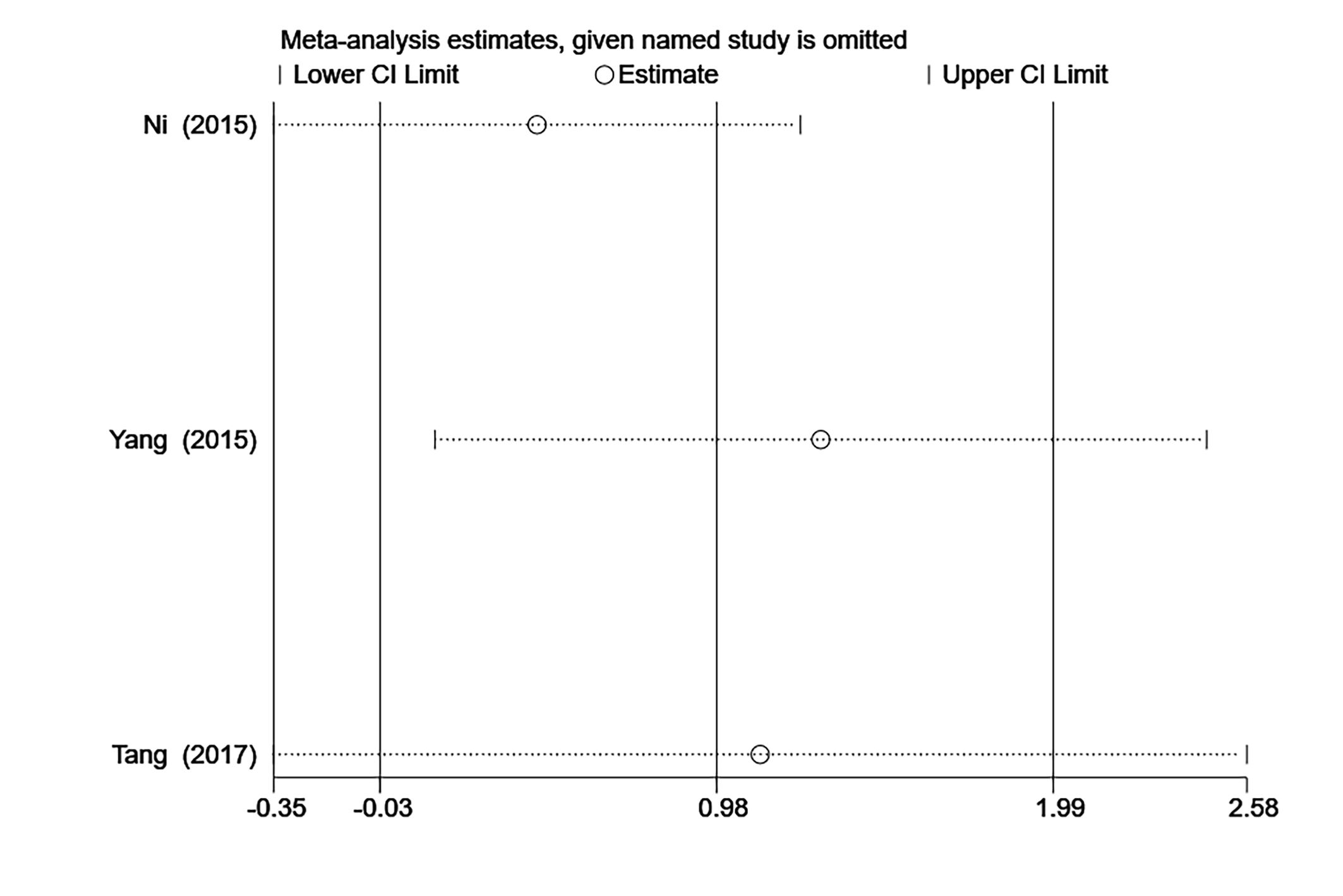


**Supplementary Figure 13.** Sensitivity analysis for the MMSE scores on post-operative 5d.


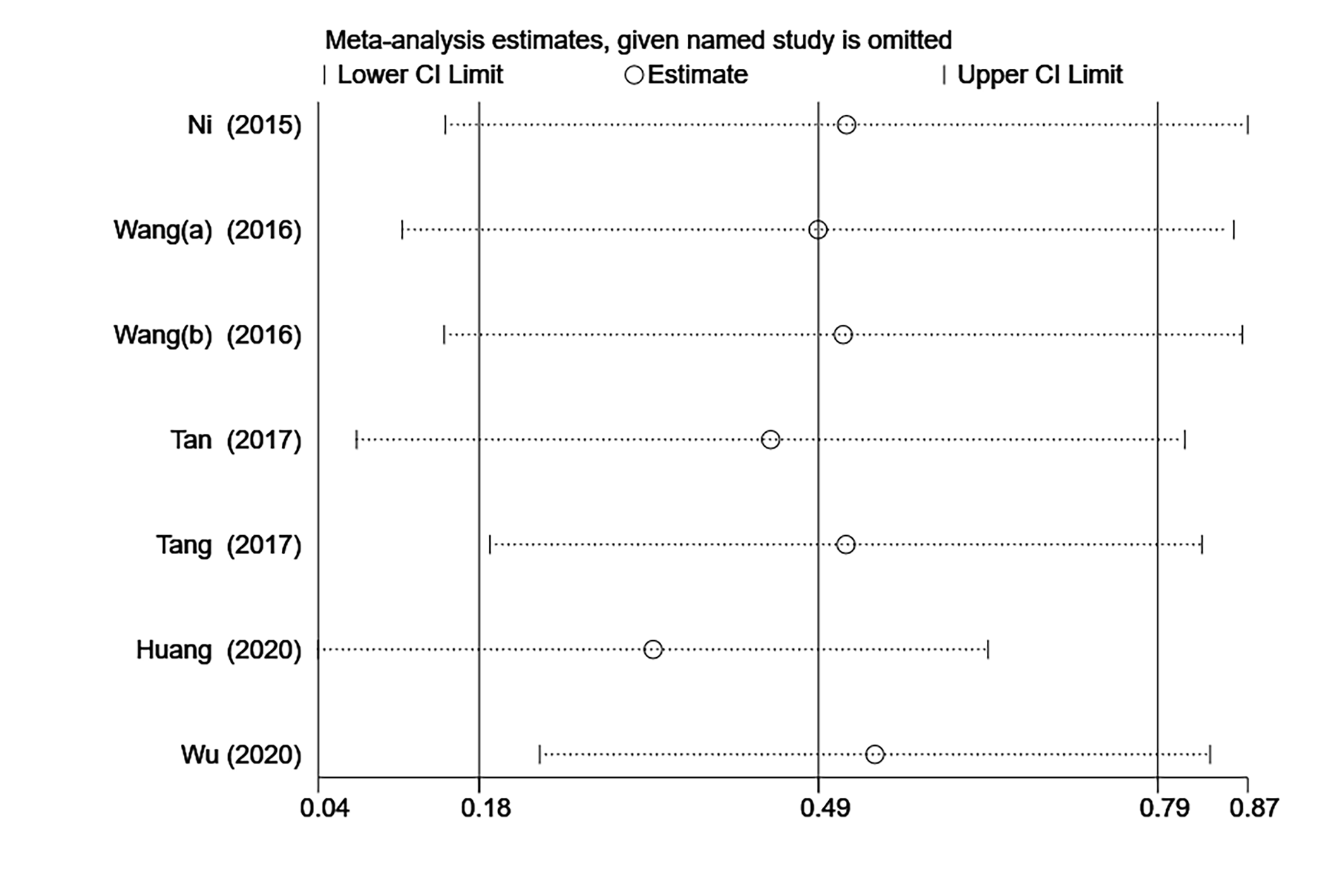


**Supplementary Figure 14.** Sensitivity analysis for the MMSE scores on post-operative 7d.


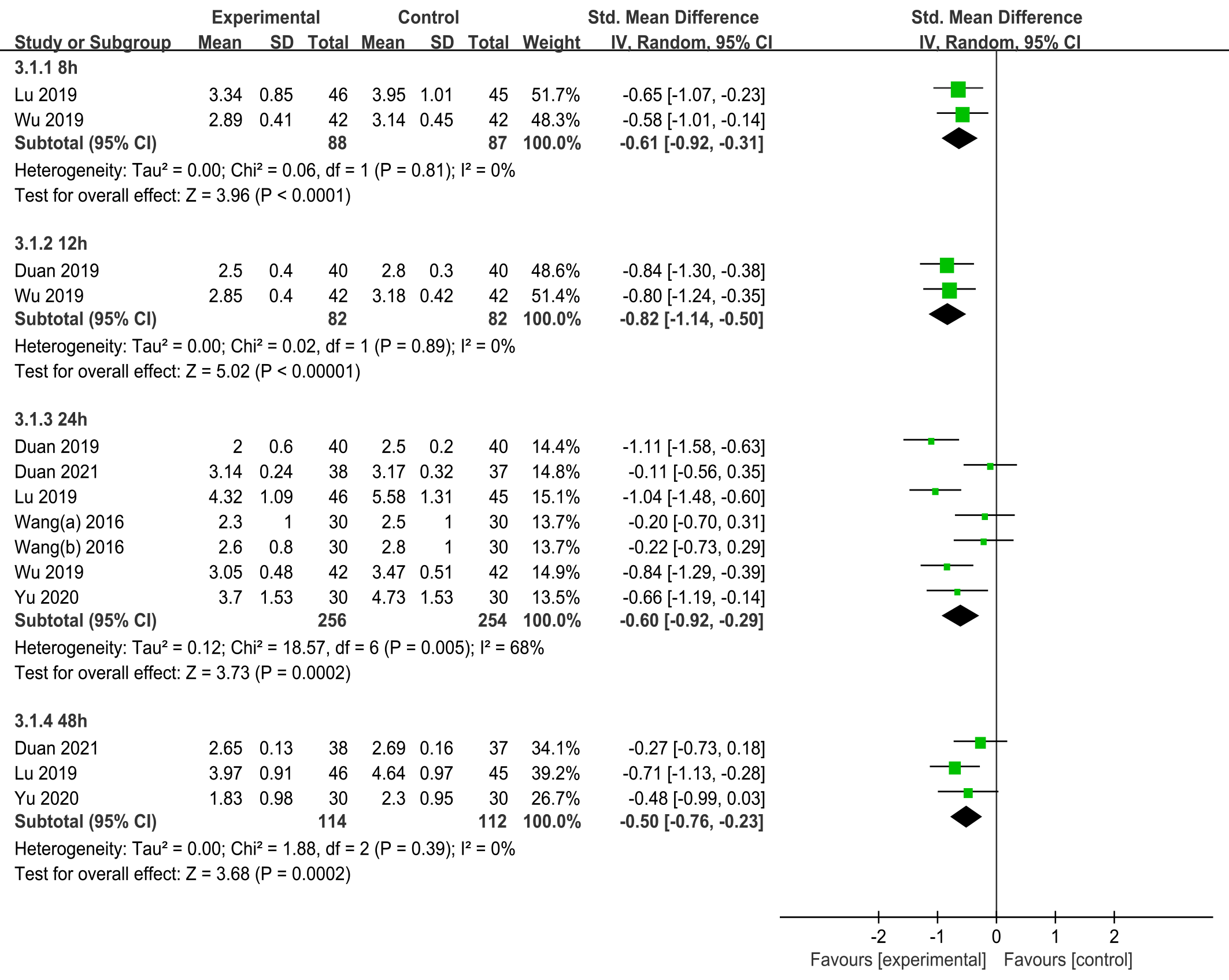


**Supplementary Figure 15.** Meta-analysis and forest plot for VAS scores at different periods after surgery.


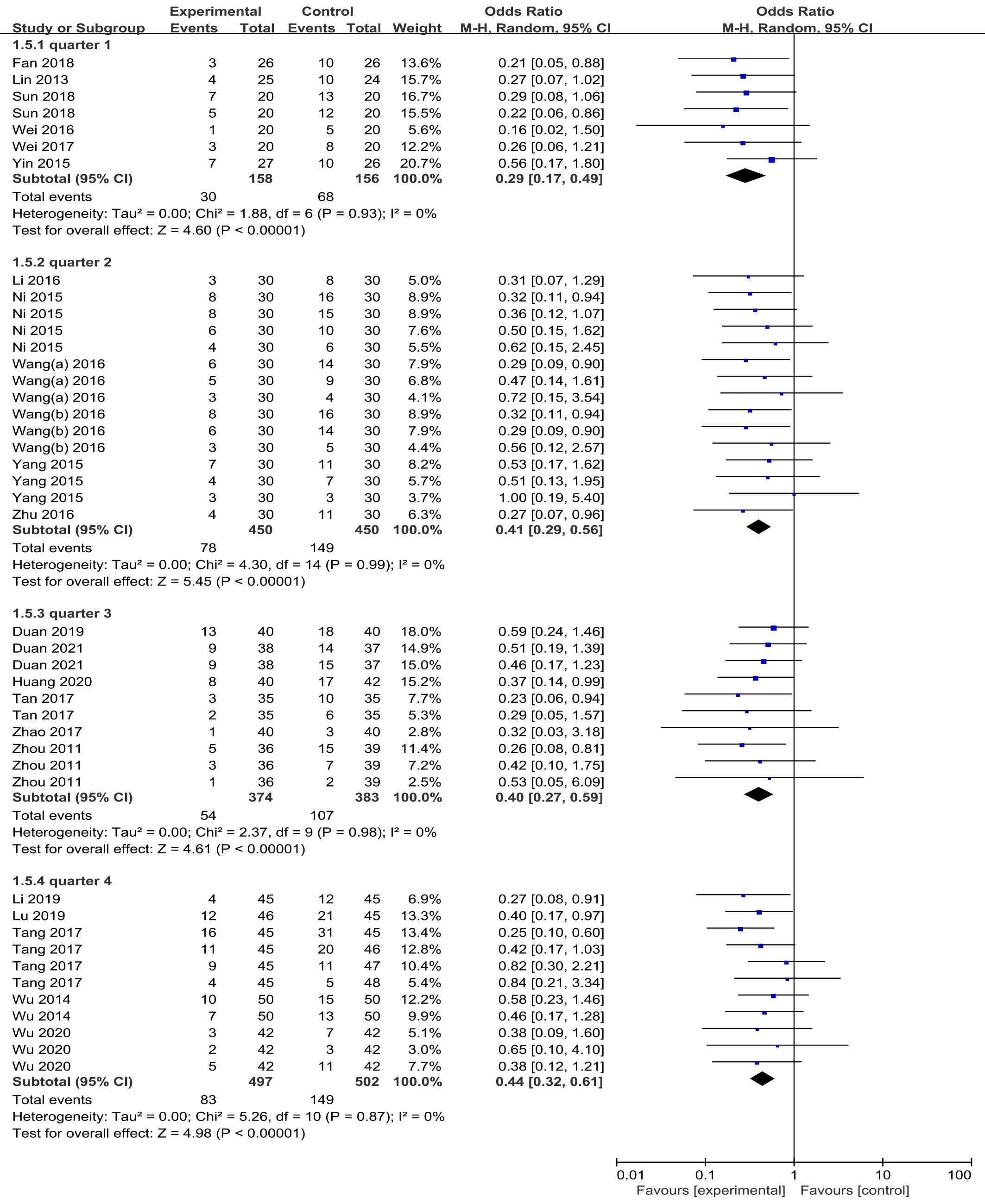


**Supplementary Figure 16.** Meta-analysis and forest plot for the incidence of POCD (subgroup analysis was grouped according to sample size).
